# Multiscale proteomic modeling reveals protein networks driving Alzheimer’s disease pathogenesis

**DOI:** 10.1016/j.cell.2025.08.038

**Published:** 2025-09-25

**Authors:** Erming Wang, Kaiwen Yu, Jiqing Cao, Minghui Wang, Pavel Katsel, Won-min Song, Zhen Wang, Yuxin Li, Xusheng Wang, Qian Wang, Peng Xu, Gefei Yu, Li Zhu, Jia Geng, Parnian Habibi, Lu Qian, Tony Tuck, Aiqun Li, Julia TCW, Panos Roussos, Kristen J. Brennand, Vahram Haroutunian, Erik C.B. Johnson, Nicholas T. Seyfried, Allan I. Levey, David A. Bennett, Junmin Peng, Dongming Cai, Bin Zhang

**Affiliations:** 1 Department of Genetics and Genomic Sciences, Icahn School of Medicine at Mount Sinai, One Gustave L. Levy Place, New York, NY 10029, USA; 2 Mount Sinai Center for Transformative Disease Modeling, Icahn School of Medicine at Mount Sinai, One Gustave L. Levy Place, New York, NY 10029, USA; 3 Icahn Genomics Institute, Icahn School of Medicine at Mount Sinai, One Gustave L. Levy Place, New York, NY 10029, USA; 4 Departments of Structural Biology and Developmental Neurobiology, St. Jude Children’s Research Hospital, Memphis, TN 38105, USA; 5 Alzheimer’s Disease Research Center, Icahn School of Medicine at Mount Sinai, New York, NY 10029, USA; 6 Department of Neurology, Icahn School of Medicine at Mount Sinai, New York, NY 10029, USA; 7 Nash Family Department of Neuroscience, Icahn School of Medicine at Mount Sinai, 1425 Madison Avenue, New York, NY 10029, USA; 8 Department of Psychiatry, Icahn School of Medicine at Mount Sinai, 1425 Madison Avenue, New York, NY 10029, USA; 9 MIRECC, Peters VA Medical Center, 130 West Kingsbridge Road, Bronx, New York, NY 10468, USA; 10 Center for Proteomics and Metabolomics, St. Jude Children’s Research Hospital, Memphis, TN 38105, USA; 11 Department of Neurology, University of Tennessee Health Science Center, Memphis, TN 38163, USA; 12 Department of Pharmacology, Physiology & Biophysics, Boston University Chobanian and Avedisian School of Medicine, Boston, MA 02118, USA; 13 Bioinformatics Program, Boston University Faculty of Computing & Data Sciences, Boston, MA 02215, USA; 14 Department of Psychiatry, Yale University School of Medicine, New Haven, CT 06511, USA; 15 Goizueta Alzheimer’s Disease Research Center, Department of Neurology, Emory University School of Medicine, Atlanta, GA, USA; 16 Department of Biochemistry, Emory University School of Medicine, Atlanta, GA, USA; 17 Rush Alzheimer’s Disease Center, Rush University Medical Center, Chicago, IL, USA; 18 Department of Neurology, University of Minnesota, Minneapolis, MN 55455, USA; 19 N. Bud Grossman Center for Memory Research and Care, University of Minnesota, Minneapolis, MN 55455, USA; 20 Geriatric Research Education & Clinical Center (GRECC), Minneapolis VA Health Care System, Minneapolis, MN 55417, USA; 21 These authors contributed equally; 22 Lead contact

## Abstract

The molecular mechanisms underlying the pathogenesis of Alzheimer’s disease (AD), the most common form of dementia, remain poorly understood. Proteomics offers a crucial approach to elucidating AD pathogenesis, as alterations in protein expression are more directly linked to phenotypic outcomes than changes at the genetic or transcriptomic level. In this study, we develop multiscale proteomic network models for AD by integrating large-scale matched proteomic and genetic data from brain regions vulnerable to the disease. These models reveal detailed protein interaction structures and identify putative key driver proteins (KDPs) involved in AD progression. Notably, the network analysis uncovers an AD-associated subnetwork that captures glia-neuron interactions. AHNAK, a top KDP in this glia-neuron network, is experimentally validated in human induced pluripotent stem cell (iPSC)-based models of AD. This systematic identification of dysregulated protein regulatory networks and KDPs lays down a foundation for developing innovative therapeutic strategies for AD.

## In brief

Multiscale proteomic network modeling of Alzheimer’s disease integrates large-scale proteomic and genetic data from vulnerable brain regions and reveals key driver proteins such as AHNAK within a glia-neuron subnetwork. These findings offer valuable insights into disease mechanisms and highlight potential therapeutic targets.

## Graphical Abstract

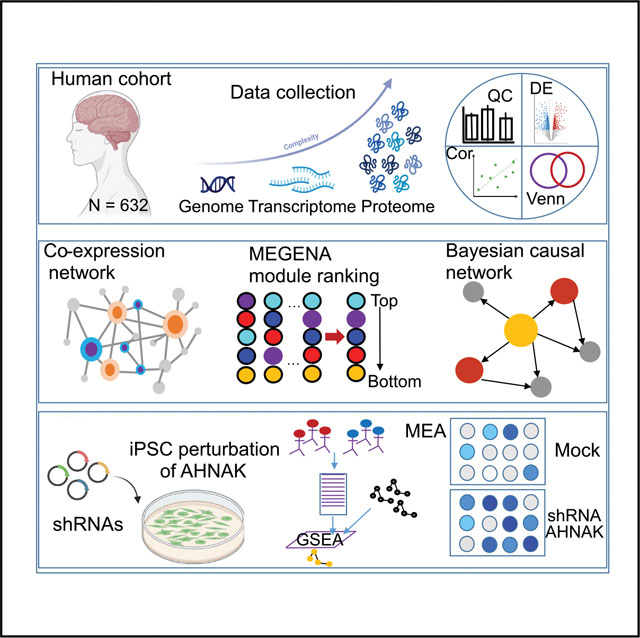

## INTRODUCTION

Alzheimer’s disease (AD) is the most prevalent form of dementia.^1,2^ While a few compounds, for example, lecanemab^3–5^ and aducanumab,^6,7^ have shown modest efficacy in reducing β-amyloid peptides (Aβ) in the brain and slowing cognitive decline in people with early AD, the majority of the experimental drugs for AD have failed.^8,9^ Pathologically, AD is characterized by the accumulation of neurofibrillary tangles, dystrophic neurites, abundant extracellular fibrils of Aβ, and inflammation.^10^ However, the etiology of sporadic AD, especially late-onset AD (LOAD), remains poorly understood.^1,2,10^

Multiomics, such as genomics,^11–13^ methylomics,^14–16^ transcriptomics,^17–19^ proteomics,^20–23^ and metabolomics,^24^ coupled with network biology approaches,^10,25^ have shown great potential to dissect key pathways and drivers for complex diseases, including AD.^26^ Proteomics is particularly important since alterations in protein expression are more directly linked to phenotypic variations than changes at the genetic or transcriptomic level.^27^ To date, only a limited number of proteomic studies of human brain tissues have been conducted for AD.^20,21,23,28–32^ Currently, large-scale deep proteomic profiling and advanced network biology modeling of the parahippocampal gyrus (PHG), the brain region most susceptible to AD,^18,33^ remain unavailable. The PHG encompasses most of the perirhinal, entorhinal, and posterior parahippocampal cortices, residing within the medial temporal lobe. It is essential for memory formation and the recall of visual scenes. The PHG plays a pivotal role in processing contextual information related to the environment and spatial memory, serving as a relay between the hippocampus and other brain regions. Abnormalities in the PHG structure may indicate underlying conditions like AD. Findings from functional imaging analyses have consistently identified abnormal changes in the parahippocampus, such as gray matter atrophy, reduced cortical thickness, decreased white matter volume, and white matter microstructural abnormalities, which are related to cognitive compromise in AD/mild cognitive impaired (MCI) patients.^34–36^

To comprehensively understand the molecular dysregulation in the PHG of AD brains and ultimately enable the discovery of distinct mechanisms of AD, in this study we performed deep profiling of the proteome of the PHG from 198 postmortem AD, MCI, and normal control (NL) brains in the Mount Sinai/JJ Peters VA Medical Center Brain Bank (MSBB).^11,18,33,37^ We then conducted a multiscale protein network analysis, combining protein co-expression and Bayesian causal network approaches, to systematically identify key protein subnetworks and drivers of AD by integrating the matched genetic, proteomic, clinical, and pathological data. The multiscale network analysis revealed an AD-associated subnetwork that captures the interactions among neurons, microglia, and astrocytes, and such interactions were validated by the so-far largest single-nucleus RNA sequencing (snRNA-seq) dataset in AD. We further validated the functional role of AHNAK, one of the top protein drivers of the glia-neuron network in AD, using human induced pluripotent stem cell (iPSC)-derived astrocytes and their co-cultured mouse neurons. This study presents the important set of multiscale proteomic regulatory networks and the inaugural list of putative causal protein drivers of AD, laying a foundation for systematically uncovering molecular mechanisms underlying AD and advancing the development of next-generation therapeutics for AD.

## RESULTS

### Overview of the study design

**Figure 1.**
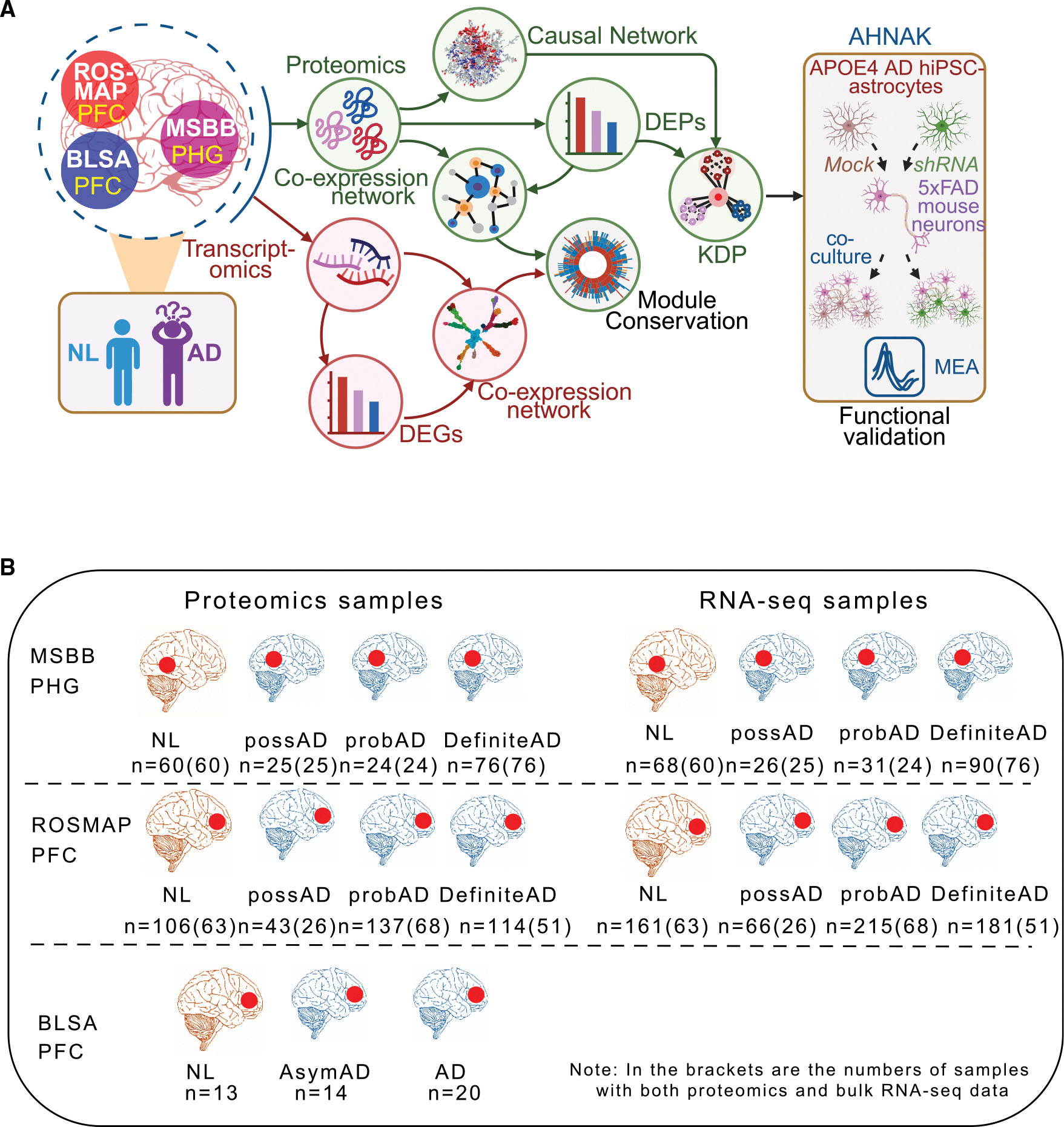
Flowchart illustrating the computational and experimental workflow of the study (A) The workflow of this study includes generation and curation of multiomic cohorts in AD, identification of differentially expressed proteins (DEPs), construction and analysis of protein co-expression and causal networks, prediction of driver proteins, and experimental validation of a key driver protein. (B) Sample tissue collection. The left and right panels show proteomics profiling and bulk RNA-seq, respectively. In the MSBB and ROSMAP cohorts, AD status was classified by the CERAD: NL, normal control; possAD, possible AD; probAD, probable AD; DefiniteAD, definite AD. *n* indicates the number of samples in each group, and the number in a bracket is the number of brains with both proteomic and bulk RNA-seq data. In the BLSA cohort, the AD status was classified as AD, AsymAD (pathologically diagnosed as AD but without dementia) cases, and NL. PHG, parahippocampal gyrus; PFC, prefrontal cortex. See also Table S1, pages 1–5.

This study focuses on the proteomic data in the two large multiomic AD cohorts, MSBB^11,18,33,37^ and the longitudinal Religious Orders Study/Memory and Aging Project (ROSMAP)^28,29,38,39^ (Figure 1). Note that the multiomic data in ROSMAP were generated from the samples in the dorsal lateral prefrontal cortex (PFC). In addition to the differential protein expression and protein-trait correlation analyses, we performed the protein co-expression network analysis to identify co-expressed protein modules at multiple scales of compactness, followed by the protein causal network analysis to predict putative protein drivers of AD (Figure 1A). AHNAK, one of the top protein drivers in the protein module most associated with AD, was experimentally validated using human iPSC models of AD. As both cohorts include matched transcriptomic data in multiple brain regions, we also assessed the conservation of gene and protein modules within the same region and between different regions, leading to the identification of PHG and proteome-specific protein modules associated with AD.

### Development of a cohort of proteomic data from 185 samples of the PHG from AD and non-AD brains

We performed proteomic profiling of the PHG of 185 postmortem AD and non-AD brains from the MSBB cohort. The PHG is among the brain regions most vulnerable to AD,^11,18,37^ and thus understanding its proteome would help to gain more relevant insights into AD pathogenesis. The proteomic data were preprocessed by our in-house pipeline for sample quality control (QC) in multiomics assays^11,20^ (also see the details in the STAR Methods), and 185 samples passed the QC. Based on the consortium to establish a registry for Alzheimer’s disease (CERAD) neuropathological classification,^18^ the QC-ed proteomic data include 76 definite, 24 probable, and 25 possible AD cases, as well as 60 healthy NL, respectively (Figure 1B, top left). There were 116 females and 69 males with an average age at death of 84.5 years old, ranging from 61.0 to 108.0 years (Table S1, pages 1 and 2). The AD donors were selected to exclude donors with non-AD comorbidities.

We identified a total of 12,147 unique protein isoforms (here-after referred to as proteins, unless otherwise stated) that had detectable signals across each of the 185 brain samples. These protein isoforms are generated from 9,272 unique genes (1.34 protein isoforms per gene on average). The normalized expression levels of the proteins were then adjusted for the covariates such as sex, race, age at death, postmortem interval (PMI), and batch (STAR Methods).

### Identification of AD-associated proteins

To facilitate the identification of AD-associated proteins, we stratified the subjects into 3 groups that represent disease severity stages from normal through moderate to high severity in AD pathology and dementia with respect to each AD neuropathological or clinical trait.^18,33,40,41^ Specifically, for Braak score (BS), subjects were classified into low (BS ≤ 2), medium (2 < BS ≤ 4), and high (BS > 4) groups; for the clinical dementia rating (CDR), subjects were stratified into cognitive normal (nondemented) (CDR = 0), impaired (0 < CDR ≤ 2), and demented (CDR > 2) groups; for plaque mean density (PMD), subjects were classified into normal (PMD = 0), mild (0 < PMD ≤ 9), and severe (PMD > 9) groups; and for CERAD score, subjects were classified into DefiniteAD (CERAD = 2), IndefiniteAD that includes probable AD (CERAD = 3) and possible AD (CERAD = 4), and NL (CERAD = 1) (Table S1, pages 1 and 2).

**Figure 2.**
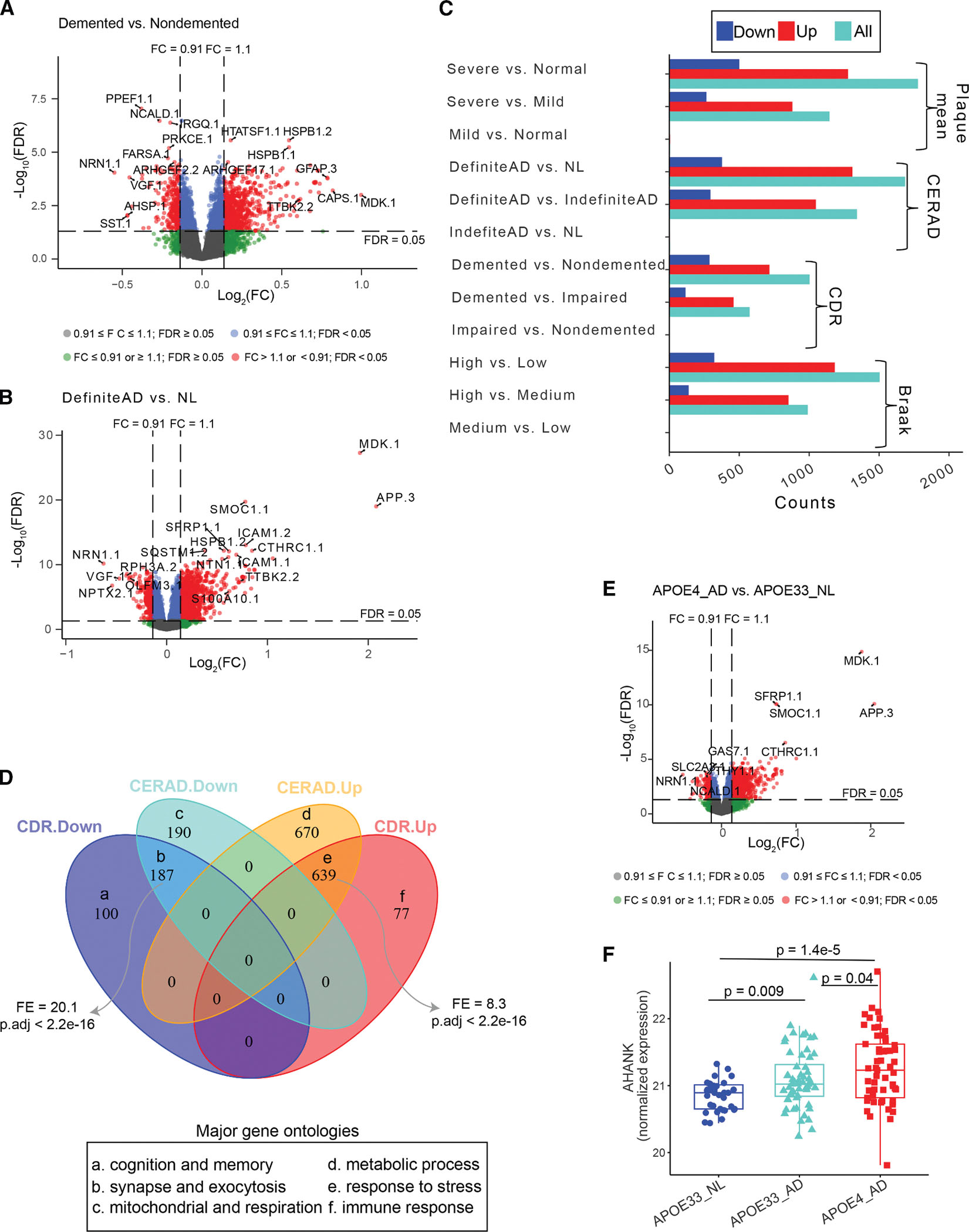
The analysis of DEPs in the MSBB cohort (A and B) Volcano plot showing DEPs in the PHG between demented vs. nondemented subjects by CDR (A) and between DefiniteAD vs. NL subjects by CERAD (B). The digit after each protein name denotes a protein isoform. NS, not significant; FC, fold change. (C) Number of DEPs with respect to different traits. (D) Intersections among the CDR- and CERAD-based DEP signatures. (E) Volcano plot showing DEPs in AD with APOE4 vs. NL with APOE33. (F) Boxplot showing the AHNAK expression across different APOE genotypes in relevance to AD pathology. See also Figure S1 and Table S2, pages 1 and 3.

We then investigated the association of each protein with the four AD neuropathological and clinical traits, including BS, CDR, CERAD, and PMD (Figures 2A and 2B; Table S2, page 1). Figure 2C summarizes the differentially expressed proteins (DEPs) identified from the pairwise comparisons among the previously defined three subject subgroups with respect to each AD trait. We have further split a DEP signature from each comparison into a downregulated and an upregulated subset. A DEP is considered upregulated if the fold change (FC) between the compared subgroups is positive; otherwise, it is deemed downregulated.

We identified over 1,000 DEPs from the comparisons of severe AD pathology vs. healthy control with respect to the clinical and neuropathological traits (e.g., DefiniteAD vs. NL) (Figure 2C; Table S2, page 1), accounting for ~10% of the total proteins evaluated. Interestingly, >70% of the DEPs were upregulated in the cases with severe AD pathology (Figure 2C). Similar trends were observed for the comparison of severe and moderate AD pathology (for example, DefiniteAD vs. IndefiniteAD) (Figure 2C). However, we detected no or a limited number of DEPs between moderate AD pathology and healthy control (Figure 2C; see discussion). Similar results were found from the correlation analysis of protein expression and the clinical and neuropathological traits of AD (Data S1, page 1; Table S2, page 2). Thus, a substantial number of proteins were differentially expressed between AD and healthy control.

To reveal the biological relevance of the DEPs in AD neuropathology, we investigated the known functional pathways enriched in these DEP signatures. As shown in Figure S1A, the DEP signatures were enriched for Gene Ontology (GO) pathways important in AD pathogenesis, such as neuronal and synapse functions in the downregulated DEP signatures and immune response in the upregulated DEP signatures (Table S2, page 3). These observations were consistent with our previous finding that downregulated DEP signatures were enriched in neuronal cells, while the upregulated DEP signatures were enriched in microglia and astrocytes (Data S1, page 2A; Table S2, page 4).^42^ We further observed significant enrichment of both AD risk genes and Aβ pathway genes in the DEP signatures (Data S1, page 2B; Table S2, page 5). These results demonstrate that the DEP signatures capture some critical components of AD neuropathology.

### CDR and CERAD capture distinct aspects of AD pathology

CDR and CERAD are two traits used to assess the progression of dementia and AD pathology, with CDR evaluating dementia severity and cognitive status and CERAD indicating case-control status for AD based on neuropathological criteria.^33,41^ In the MSBB, CDR, and CERAD were moderately correlated (*r* = 0.6, *p* = 1.1e–19),^41^ suggesting their shared and distinct association with AD progression. Therefore, we compared the protein expression signatures with respect to CDR and CERAD. While a significant number of the CDR-based DEPs overlap the CERAD-based ones (Figure 2D, top), there are a number of DEPs that are uniquely associated with CDR or CERAD (Figure 2D, top), indicating that some abnormally expressed proteins that contribute to cognitive compromise in AD are not directly associated with the cardinal neuropathological lesions.

Next, we performed GO enrichment analysis on these DEP signatures to identify associated functional pathways. Figure 2D (bottom) showed the most enriched pathway for each of the six DEP subsets in the Venn diagram, highlighting their distinct roles in AD pathology and dementia. For example, the DEPs that are downregulated in subjects with high CDR scores are enriched for biological processes such as cognition, learning, or memory^43–48^ (Figure 2D, bottom; Table S2, page 6).

### *APOE* genotypes are critical in AD-associated proteome

*APOE* genotype is a major risk factor of AD, with the *APOE* ε4/ε4 (*APOE* 44) genotype exerting particularly detrimental effects. Therefore, we assessed the impact of different *APOE* genotypes on the proteome in connection with AD pathology. As only 4 of the 185 subjects in the MSBB carry the *APOE* 44 genotype, we combined the subjects with *APOE* ε4/ε4 and *APOE* ε3/ε4 as a single category termed “APOE4.” Based upon CERAD, we further combined DefiniteAD and IndefiniteAD as an AD in contrast to the NL group. We then focused on the following three subgroups as the combinations of *APOE* genotypes and disease statuses: APOE4-AD (57 subjects), APOE33-AD (56 subjects), and APOE33-NL (35 subjects). We then identified DEPs for the comparisons of APOE4-AD vs. APOE33-AD, APOE4-AD vs. APOE33-NL, and APOE33-AD vs. APOE33-NL (Table S2, page 7). We detected 244 down- and 1,114 upregulated DEPs between APOE4-AD and APOE33-NL (Figure 2E; Table S2, page 7) but only 42 DEPs between APOE33-AD and APOE33-NL. Interestingly, two DEPs (NSUN5P2 and GPM6A) showed opposite directions of differential expression in APOE33-AD and APOE4-AD when compared with APOE33-NL. There is no DEP between APOE4-AD and APOE33-AD (Table S2, page 7). As an example, we showed the progressive increase in the protein expression of AHNAK from APOE33-NL to APOE33-AD and then APOE4-AD (Figure 2F). These results suggested the potential interaction between *APOE*4 genotypes and disease status, which might aggravate the progression of AD pathology.

### Sex-shared and specific AD-associated proteome

Given that sex is a major risk factor in AD pathology,^49^ we performed differential expression analysis on sex-stratified AD groups (see the STAR Methods for details). In males, we identified 142 downregulated and 434 upregulated DEPs in DefiniteAD compared with NL, and 220 downregulated and 784 upregulated DEPs in DefiniteAD vs. NL in females (Table S2, page 8). Although there is a significant overlap between the male and female DEP signatures, a substantial number of DEPs are unique to each sex (Figure S1B), indicating pronounced sex-specific differences in proteomic changes associated with AD.

### DEP signatures are replicated in independent AD proteomic datasets and AD mouse model

**Figure 3.**
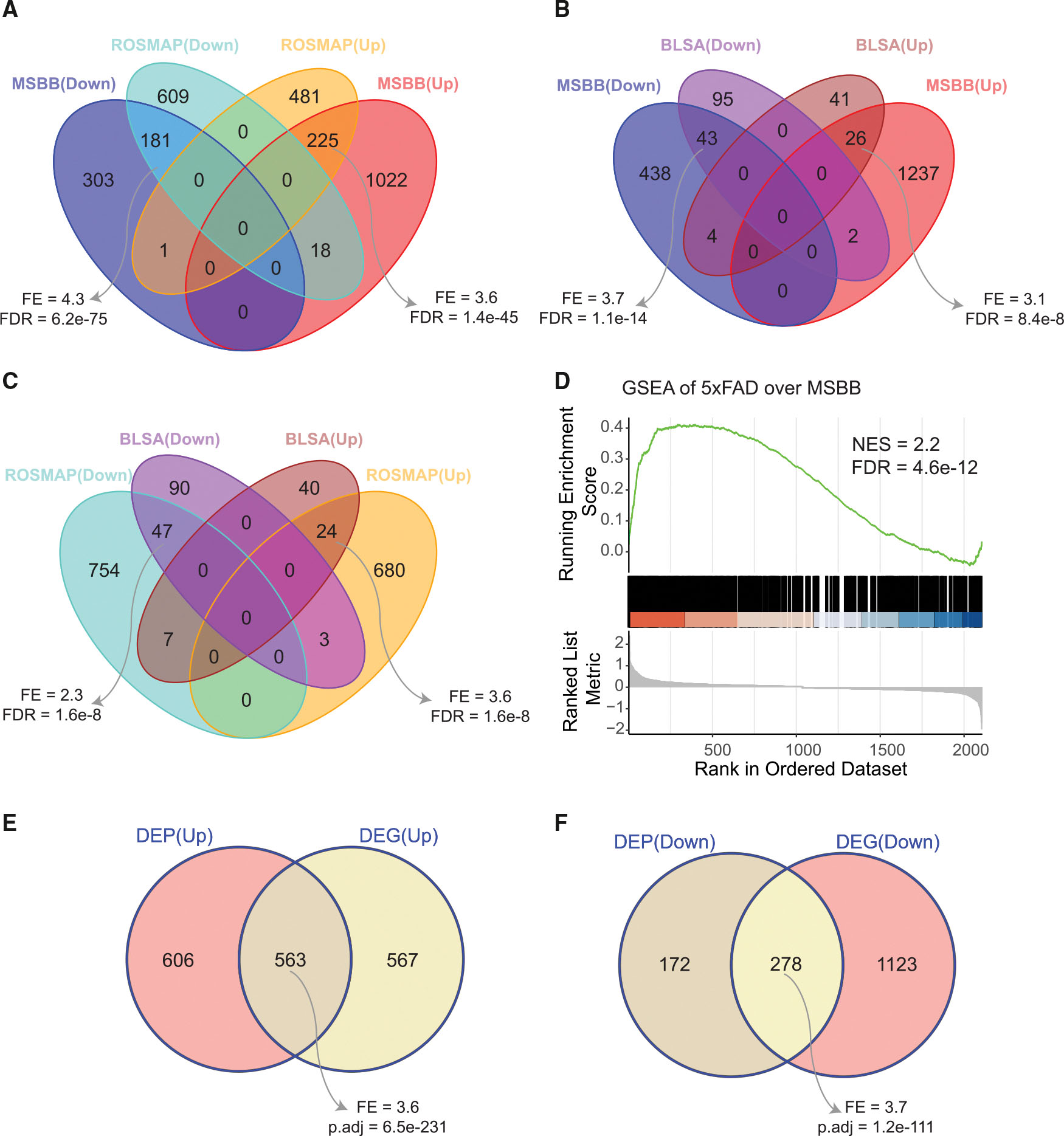
Independent validation of the DEP signatures in the MSBB cohort using the ROSMAP and BLSA cohorts (A) Intersections between the MSBB and ROSMAP DEP signatures. (B) Intersections between the MSBB and BLSA DEP signatures. (C) Intersections between the ROSMAP and BLSA DEP signatures. (D) The gene set enrichment analysis (GSEA) plot showing the enrichment trend of the 5xFAD DEPs over an MSBB DEP signature. NES, normalized enrichment score. FDR is BH-adjusted *p* value. (E and F) Intersections between the upregulated DEP and DEG signatures (E) and downregulated DEP and DEG signatures (F) in the MSBB cohort. The DEP and DEG signatures were based on the four clinical traits as described in the main text. See the STAR Methods for details. In (A)–(C), FE stands for fold enrichment. FDR is the Benjamini & Hochberg (BH)-adjusted *p* value derived from the hypergeometric test. See also Figure S2; Table S1, page 1; and Table S3, pages 1–6.

To evaluate the aforementioned key findings, we examined how the DEP signatures from the MSBB proteomics data (Table S2, page 1) would overlap those from the independent proteomic datasets generated from the PFC in the ROSMAP^28,29,38,39^ (Table S3, page 1; Figure 1B, middle left) and Baltimore Longitudinal Study on Aging (BLSA)^50–52^ cohorts (Table S3, page 2; Figure 1B, bottom left). To facilitate the comparison across the three different cohorts, we generated a final DEP signature as the union of the DEPs (STAR Methods) with respect to individual traits in each cohort. The resulting three final DEP signatures significantly overlap each other (Figures 3A–3C). For example, the down- and upregulated DEP signatures in the MSBB cohort significantly overlap the down- (fold enrichment [FE] = 4.3, false discovery rate [FDR] = 6.2e–75) and upregulated DEPs (FE = 3.6, FDR = 1.4e–45) in the ROSMAP cohort, respectively (Figure 3A).

We also examined if our AD proteomic signatures overlap those from an AD mouse model. The proteomes of 5xFAD and its counterpart wild-type (WT) mice at the age of 6 months were profiled and processed as described^20^ (STAR Methods). The decision to evaluate DEPs in 5xFAD vs. WT mice at 6 months of age was based on evidence that memory function in 5xFAD mice begins to deteriorate around 5 months,^53^ as assessed by Y-maze and Morris water maze.^54–56^ There are 227 DEPs between 5xFAD and WT mice at the age of 6 months based on the multiple testing corrected *p* value (FDR < 0.05) (Table S3, page 5). Since a small number of DEPs were identified in 5xFAD mice by FDR < 0.05, we relaxed the criterion by using a nominal *p* value < 0.05. Gene set enrichment analysis (GSEA) shows that there is a strong enrichment between the MSBB AD DEP signature and the mouse AD DEP signature (normalized enrichment score [NES] = 2.2, adjusted *p* = 4.6e–12) (Figure 3D).

Finally, we examined the concordance between the DEP and differentially expressed gene (DEG) signatures^33^ (STAR Methods) in the PHG from the MSBB cohort^33^ (Figure 1B, top right). It is observed that 48.2% of the upregulated DEPs and 79.2% of the downregulated DEPs were consistently up- and downregulated in AD at the mRNA level, respectively (Figures 3E, 3F, and S2A; STAR Methods). As expected, the protein levels of these conserved DEPs were also significantly correlated with their corresponding mRNA profiles (Figures S2B–S2D) in both the MSBB and ROSMAP cohorts.

Together, these results demonstrate that our human DEP signatures are not only highly reproducible but also strongly associated with AD pathogenesis.

### Protein co-expression network analysis reveals protein modules associated with AD

**Figure 4.**
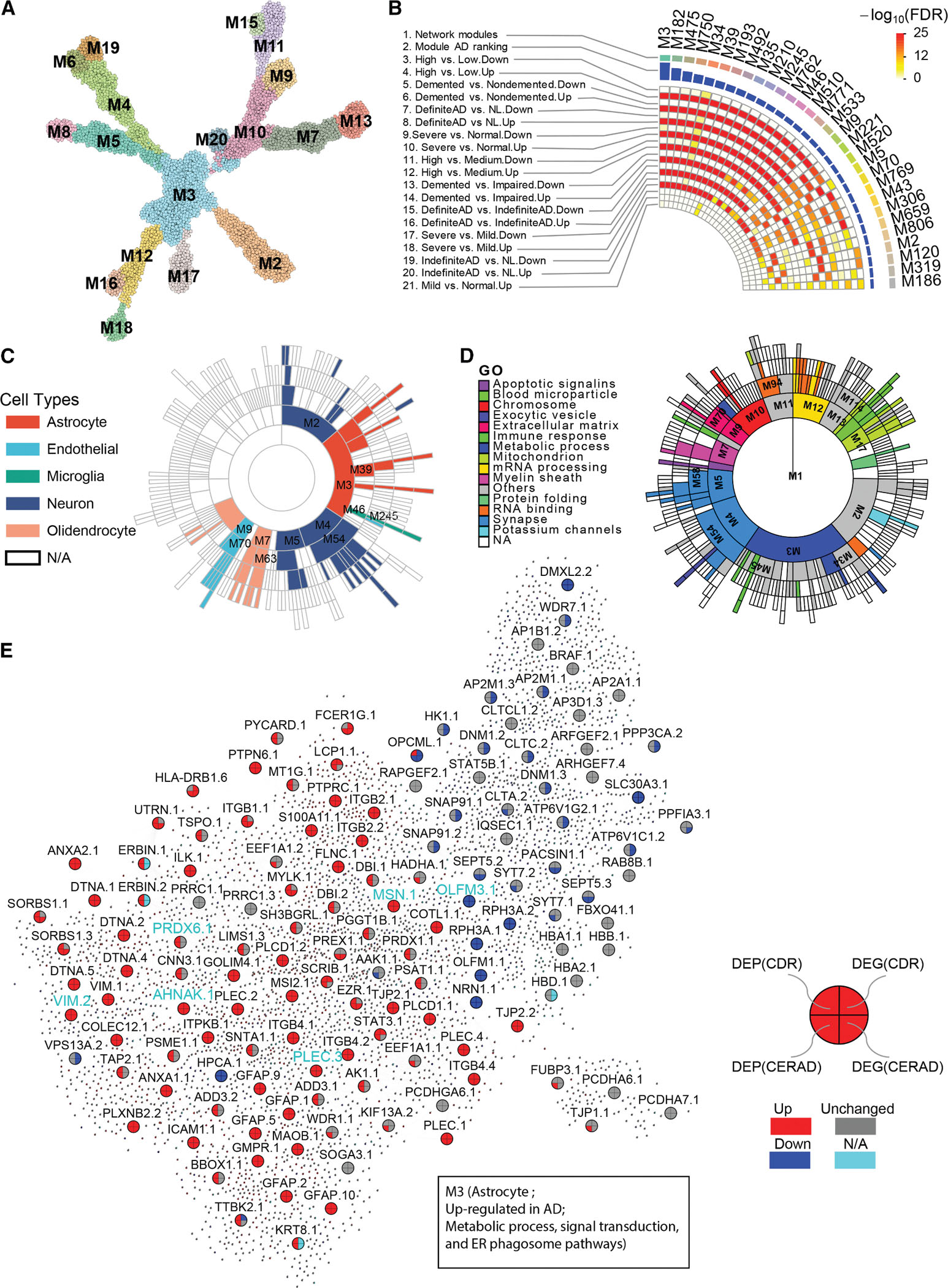
Protein co-expression network analysis of the PHG proteomics in the MSBB (A) The global protein co-expression network highlighting the top hierarchical modules. (B) Circos plot of the 30 top-ranked modules. Track 1 represents the top 30 modules in the descendant order of the relevance to AD. Track 2 denotes the relevance scores of the modules computed as the sum of the adjusted *p* values of the DEP signatures shown in tracks 3–21. (C) Cell-type specificity of the protein modules based on the most enriched brain cell-type marker gene signature.^58^ Highlighted are the modules with the best enrichment FDR less than 0.05. (D) Major GO pathways associated with the MEGENA modules (FDR < 0.05). (E) The MEGENA subnetwork for M3. Each node circle was divided into four sections. The left and right half circles represent protein and gene differential expression statuses with respect to CDR and CERAD, respectively. NA means that there is no matched DEG. KDPs are highlighted as large nodes. See also Figure S3 and Table S4, pages 1–6.

To investigate the structures of complex interactions among the proteins in AD, we constructed a protein co-expression network through the multiscale embedded gene co-expression network analysis (MEGENA) of the entire profiled proteomes^57^ (Figure 4A; Table S4, pages 1–3). We identified 386 co-expressed protein modules, ranging in size from 10 to 3,118 proteins (Table S4, page 3). To identify the co-expression network modules that are associated with AD, we intersected these protein modules with the DEP signatures (Figure 2C) and rank-ordered them based on a total enrichment score (Figure 4B, track 2), calculated as the sum of significance levels from multiple enrichment tests^17,18^ (Table S4, page 4; Figure 4B, tracks 3–21).

Figure 4B shows the top 30 ranked modules, ranked in the descending order of the association strength with AD. Among the top AD-associated 30 modules, five (M5, M2, M120, and M186) are enriched for the downregulated DEP signatures, while the other 25 are enriched for the upregulated DEP signatures. These AD-associated modules are cell-type specific based on their enrichment of human brain cell-type specific marker signatures^58^ (Figure 4C; Table S4, page 5). For example, many down-regulated modules (e.g., M2, M4, M5, M54, and M58) are enriched with neuronal markers, while glia-specific upregulated modules (e.g., M3, M46, M245, M533, and M771) are enriched with astrocytic or microglial markers.^21,23,42^ These brain cell-type-specific modules are involved in diverse biological functions (Figure 4D; Table S4, page 6).^19^ For example, the top-ranked module M3 (Figure 4B), which is upregulated in AD (Figure 4E), is involved in various critical biological pathways such as metabolic process, antigen presentation, signal transduction, and endoplasmic reticulum (ER) phagosome (Figure 4D; Table S4, page 6). Thus, the co-expression network analysis highlights several functional pathways of co-expressed protein modules, which may contribute to AD pathogenesis (Figure S3). For example, M39, the sixth highest-ranked module, which functions in astrocytes, is enriched for upregulated DEPs and is involved in cytokine signaling (Figure S3A; Table S4, page 6). Many member proteins in M39 and other top modules, such as the extracellular matrix-enriched endothelial module M9 (Figure S3B), the microglia module M245 (Figure S3C), and the neuronal module M5 (Figure S3D), are consistently up- or down-regulated in AD at both the mRNA and protein levels when compared with NL.

Another key finding is that the module M3 captures the interactions among downregulated neuronal, activated astrocytic, and activated microglia proteins (Figure 4E). To validate this finding, we calculated the cell-type-specific pseudobulk data from the snRNA-seq dataset^59^ (STAR Methods) and identified 2,394 significant correlations between the four hub proteins (OPCML, OLFM3, RPH3A, and NRN1) in excitatory neurons and all of the astrocyte proteins in M3, and 294 significant correlations between the same four neuronal hub proteins and the microglia genes in M3. In inhibitory neurons, these four neuronal hub proteins had 2,497 and 342 significant interactions with the astrocyte proteins and the microglia proteins in M3, respectively. Additionally, seven astrocyte hub proteins (VIM, GFAP, PLEC, PRDX6, EZR, AHNAK, and MSN) in M3 had 1,182, 805, and 264 significant correlations with the proteins in excitatory neurons, inhibitory neurons, and microglia in M3, respectively. Together, these results revealed the intriguing interactions among neurons, astrocytes, and microglia through the proteomic subnetwork M3.

### Identification and characterization of the PHG proteomics-specific modules

**Figure 5.**
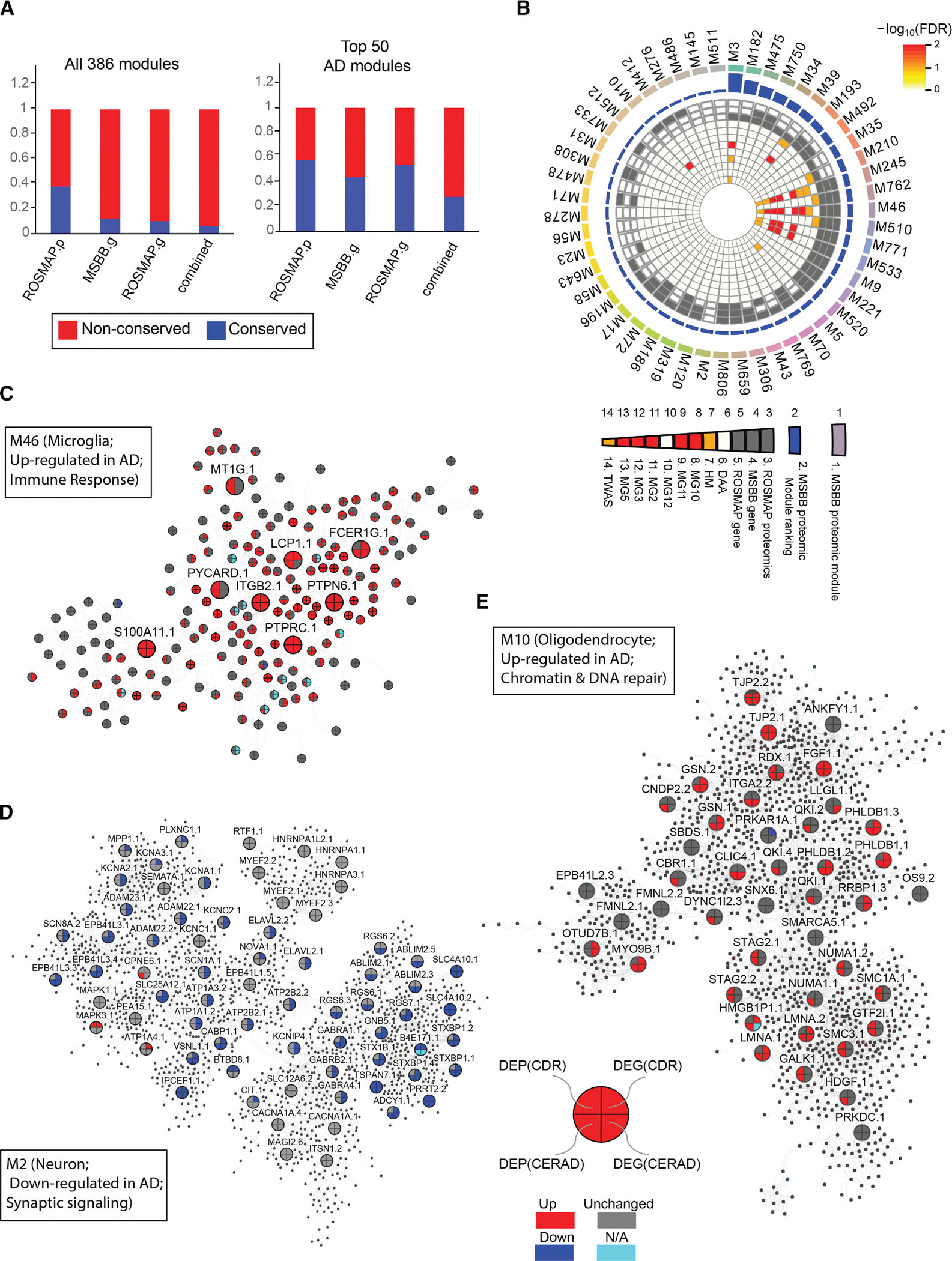
Identification and characterization of the PHG protein-specific modules associated with AD (A) Bar plots showing the number of the PHG protein modules conserved in the MSBB PHG gene co-expression network (MSBB.g), the ROSMAP-protein network (ROSMAP.p), and the ROSMAP-gene co-expression network (ROSMAP.g). The left plot is for all the PHG protein modules, and the right plot is for the top 50 AD-associated PHG protein modules. (B) Circos plot showing the conservation status and characteristics of the top 50 AD-associated protein modules in the MSBB PHG data. Tracks 3–5 show the conservation in the three gene and protein co-expression networks mentioned in (A) (gray and white blocks represent conservation and non-conservation, respectively). Tracks 6–14 show the enrichment of various signatures, including the disease-associated astrocyte (DAA) signature (6); the homeostatic microglia (HM) signature^60^ (7); the signatures of the MGs, including microglia 10 (MG10) (8), MG11 (9), MG12 (10), MG2 (11), MG3 (12), and MG5 (13); and the signature of microglia TWAS (14).^61^ The color intensity of the blocks from rings 6 and 14 is proportional to −log10(FDR) of the hypergeometric test. (C–E) Network plots of the protein module M46 (C), M2 (D), and M10 (E) in the MSBB PHG protein co-expression network. Each node circle was divided into four sections. The left and right half circles represent protein and gene differential expression statuses with respect to CDR and CERAD, respectively. NA means that there is no matched DEG. KDPs are highlighted as large nodes. See also Figure S4 and Table S5, pages 5–8.

We then performed the module conservation analysis to identify the protein-specific modules in the PHG in comparison with the gene co-expression network in the PHG of the MSBB cohort and the gene and protein co-expression networks in the PFC of the ROSMAP cohort (STAR Methods; Table S5, pages 1–4). Out of the 386 protein modules in the PHG of the MSBB cohort, 148 (38.3%), 47 (12.2%), and 40 (10.4%) modules are conserved in the ROSMAP PFC protein, the MSBB PHG gene, and the ROSMAP PFC gene networks, respectively (Figure 5A, left; Table S5, page 5). Among the top 50 AD-associated modules, 29 (58%), 22 (44%), and 27 (54%) modules are conserved in the ROSMAP PFC protein co-expression network, the MSBB PHG gene co-expression network, and the ROSMAP PFC gene co-expression network, respectively (Figures 5A, right, and S4A). Thus, the top AD-associated PHG protein modules are better conserved in the other protein and gene co-expression networks in AD.

We then proceeded to identify PHG-specific, AD-associated protein modules. Figure 5B shows the top 50 AD-associated PHG protein modules and their conservation statuses in the three aforementioned networks (tracks #3–#5, in gray and white colors). Notably, M245 and M46 are conserved across all three other networks, and a high proportion of their member proteins, in particular, the hub nodes, showed consistent differential expression at the mRNA and protein levels (see Figure S3C for the subnetwork of M245; see Figure 5C for M46). M3 and M5 are only conserved in the ROSMAP-gene network and ROSMAP-protein network, respectively. M5 is a neuron-specific module, which is downregulated in AD (Figure S3D), whereas M3 captures interactions between downregulated neuronal proteins and activated glia proteins, as previously described (Figure 4E).

Importantly, 10 modules (M2, M23, M56, M278, M476, M31, M512, M486, M145, and M511) were not conserved in any of the other 3 networks and thus are specific to the PHG protein co-expression network (Figure 5B). The neuron-specific module M2, ranked at 27^th^, is involved in chemical synaptic transmission and metal ion transport (Figure 5D; Table S5, page 6), while M31 is a child module to M2 (Table S4, page 3).

Finally, we explored the top 50 protein modules in the PHG for enriched functional pathways. Since many of the top protein modules are glia-specific, we examined their enrichment for the known gene signatures of microglia states (MGs) or disease-associated astrocytes (DAAs).^60,61^ Strikingly, the astrocytic modules M39 (Figure S3A), M210, M762, and M510 (Table S4, page 5) are enriched for the DAA gene signature (Figure 5B, track 6), while the microglia modules M245 (Figure S3C), M46 (Figure 5C), M771, and M553 (Table S4, page 5) are enriched for the gene signatures of MG states (Figure 5B, tracks 7–13). Note, M3 is a large module that is enriched not only for the astrocyte marker signature (Figure 4E) but also the MG11 and MG2 signatures (Figure 5B, tracks 9 and 11). Thus, many top AD-associated protein modules capture disease states of specific cell types.

Surprisingly, two protein modules, M5 and M10, are also enriched for the gene signatures of MG states (Figure 5B). The neuronal module M5 (Table S4, page 5) is enriched for the gene signatures of MG3 (Figure 5B, track 12). The genes shared by M5 and MG3 are mainly associated with ribosome biogenesis (Table S5, page 7). The oligodendrocyte-specific module M10 is activated in AD, as many of its member proteins are upregulated in AD (Figure 5E). M10 is enriched for the gene signature of the cell-cycle state of MG12 (Figure 5B, track 12). Indeed, the genes shared by M10 and MG12 are involved in cell-cycle signaling pathways, including chromatin structure, DNA repair, and cellular response to DNA damage (Figure S4B). DNA damage and repair have been implicated to play important roles in oligodendrocyte lineage, brain aging, and AD.^62,63^ Thus, the AD-associated modules capture cell-type-specific dysregulations in AD.

We further examined the genetics of the PHG-specific protein modules. The top AD microglia modules are enriched for the transcriptome-wide association study (TWAS) gene signature in microglia^61^ (Figure 5B, track 14), and a substantial number of the genes shared between the microglia TWAS genes and PHG-specific microglia modules are differentially expressed in AD. For example, 28% (61) of the 214 proteins shared by M3 and the microglia TWAS signature are upregulated in AD (Table S5, page 8), suggesting the potential genetic regulation of protein differential expression in AD.

### Bayesian causal protein network reveals KDPs of AD

**Figure 6.**
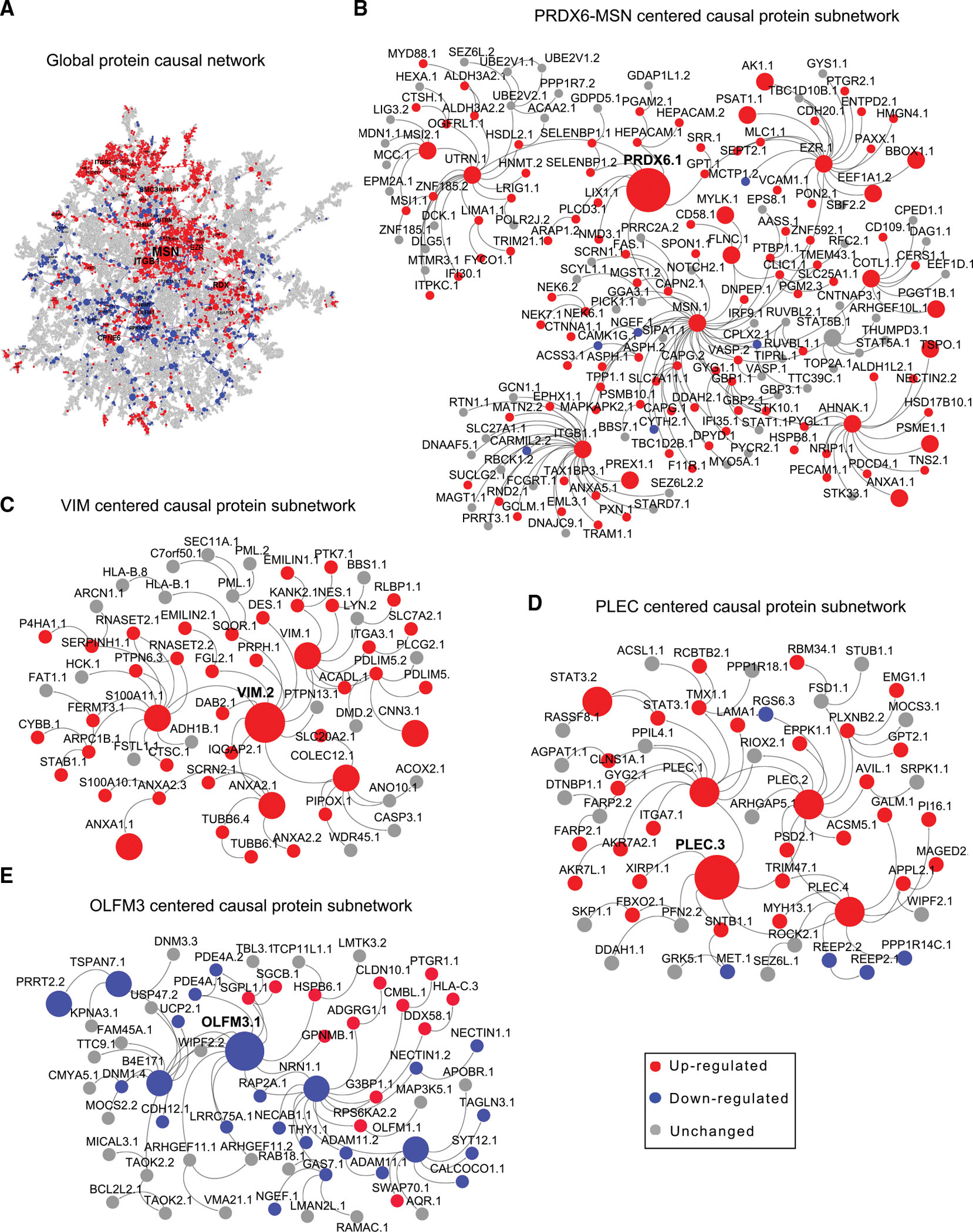
Bayesian probabilistic causal network analysis to predict putative KDPs in AD (A) The global protein Bayesian causal network based on the PHG proteomic data in the MSBB cohort. Node and font size are proportional to the link degree, whereas node color depicts the change in expression in DefinteAD vs. NL for CERAD. Red, blue, and gray stand for up, down, and unchanged, respectively. (B–E) The subnetworks centered around the top-ranked KDPs PRDX6.1 (B), VIM.2 (C), PLEC.3 (D), and OLFM3.1 (E). Node color indicates the directionality of differential expression between DefiniteAD and NL for CERAD. The nodes for KDPs are enlarged. See also Table S4, pages 7 and 8.

To uncover potential regulatory relationships among proteins, we constructed a global Bayesian causal network using the PHG proteomic data as well as the matched genetic data (details in STAR Methods; Figure 6A; Table S4, page 7), followed by key driver analysis (KDA)^17^ to identify key driver proteins (KDPs). KDA examines how the network neighborhood of each candidate protein is enriched for the previously identified AD-associated protein signatures. We identified 580 KDPs, corresponding to 471 genes (an average of 1.23 protein isoforms per gene) (Table S4, page 8). The moesin protein (MSN) and PRDX6 are the top two KDPs (Table S4, page 8), which are co-localized in a protein subnetwork that is upregulated in AD (Figure 6B). We also highlighted the causal protein subnetworks centered around VIM (Figure 6C), PLEC (Figure 6D), and OLMF3 (Figure 6E). These top-ranked KDPs have been implicated in playing a role in AD.^20–23^ For example, PRDX6 was shown to have an inhibitory effect on neurogenesis in the development of neurodegenerative diseases,^64^ attenuate ischemic oxidative damage,^65^ and anti-inflammatory processes in glia.^23^ The MSN is an essential component in the ezrin, radixin, and moesin (ERM) complex that mediates the linkage between the plasma membrane and actin cytoskeleton^66^ and was shown to be a key protein in the astrocyte/microglial metabolism module.^23,67^

Interestingly, 72% (340 out of 469) of KDPs have been understudied in AD, as they co-occur with “Alzheimer’s disease”^15^ in less than 5 papers (STAR Methods; Data S1, page 3), and 11 of these 340 proteins (AHNAK, RPH3A, EZR, PLEC, OLFM3, NUMA1, LIMS1, DTNA, PLCB1, NDUFS1, and PLCD1) are the top 30 KDPs (Table S4, pages 8 and 9; STAR Methods). Notably, 105 KDPs have not been studied in AD. Together, these results suggest that the predicted KDPs include not only well-established AD-associated targets, such as MSN,^68,69^ but also proteins that have been minimally studied or not previously investigated in the context of AD.

### Experimental validation of the protein expression of the top KDPs of AD

We verified the protein expression of the top KDPs in the PHG region from the same subjects by quantitative capillary western assays (Wes) or enzyme-linked immunosorbent assay (ELISA) (Table S6, page 1; STAR Methods). Based on the KDP ranking scores (Table S4, page 8) and the availability of antibodies, and after the collapse of isoforms from the same proteins, we selected seven top-ranked KDPs for verification, which include MSN (#1, denoting its ranking order), PRDX6 (#2), VIM (#3), AHNAK (#5), RPH3A (#6), OLFM3 (#10), and NRN1 (#14). All seven KDPs exhibited expression patterns (i.e., upregulation or downregulation) consistent with those observed in the proteomic profiling except that the downregulation of NRN1 in AD did not reach statistical significance (*p* = 0.11) (Data S1, pages 4A–4C; Table S6, page 1).

### Downregulation of AHNAK in human iPSC-derived brain cells rescues neurodegeneration

We selected AHNAK for further functional investigation for its high ranking in the KDP list and its biological significance in AD research, as it was the least studied among the top five KDPs (Table S4, page 9; STAR Methods). In both the causal and co-expression protein networks, the AD-associated DEPs are highly enriched in the AHNAK-centered subnetworks. AHNAK is upregulated in AD and significantly associated with not only neuropathological traits but also cognitive function (Table S2, page 1). AHNAK is a hub gene in the astrocytic modules M3 (Figure 4E) and M39 (Figure S3A) in the protein co-expression network in the PHG of the MSBB cohort, and its MEGENA network neighborhood is only enriched for astrocytic markers (FE = 2.7, adjusted *p* = 8.0e–26). AHNAK is also a hub gene in the module M34 of the protein co-expression network in the PFC of the ROSMAP cohort (Table S5, page 2), which is most enriched for the astrocytic markers (FE = 3.5, adjusted *p* = 8.4e–15). In addition, the snRNA-seq data in the PFC revealed that *AHNAK* is primarily expressed in astrocytes (Data S1, page 5; STAR Methods). These results strongly implicate AHNAK functions in astrocytes. Therefore, we proceeded with experimental validation studies to determine the functional roles of *AHNAK* in AD using human iPSC-derived astrocytes.

**Figure 7.**
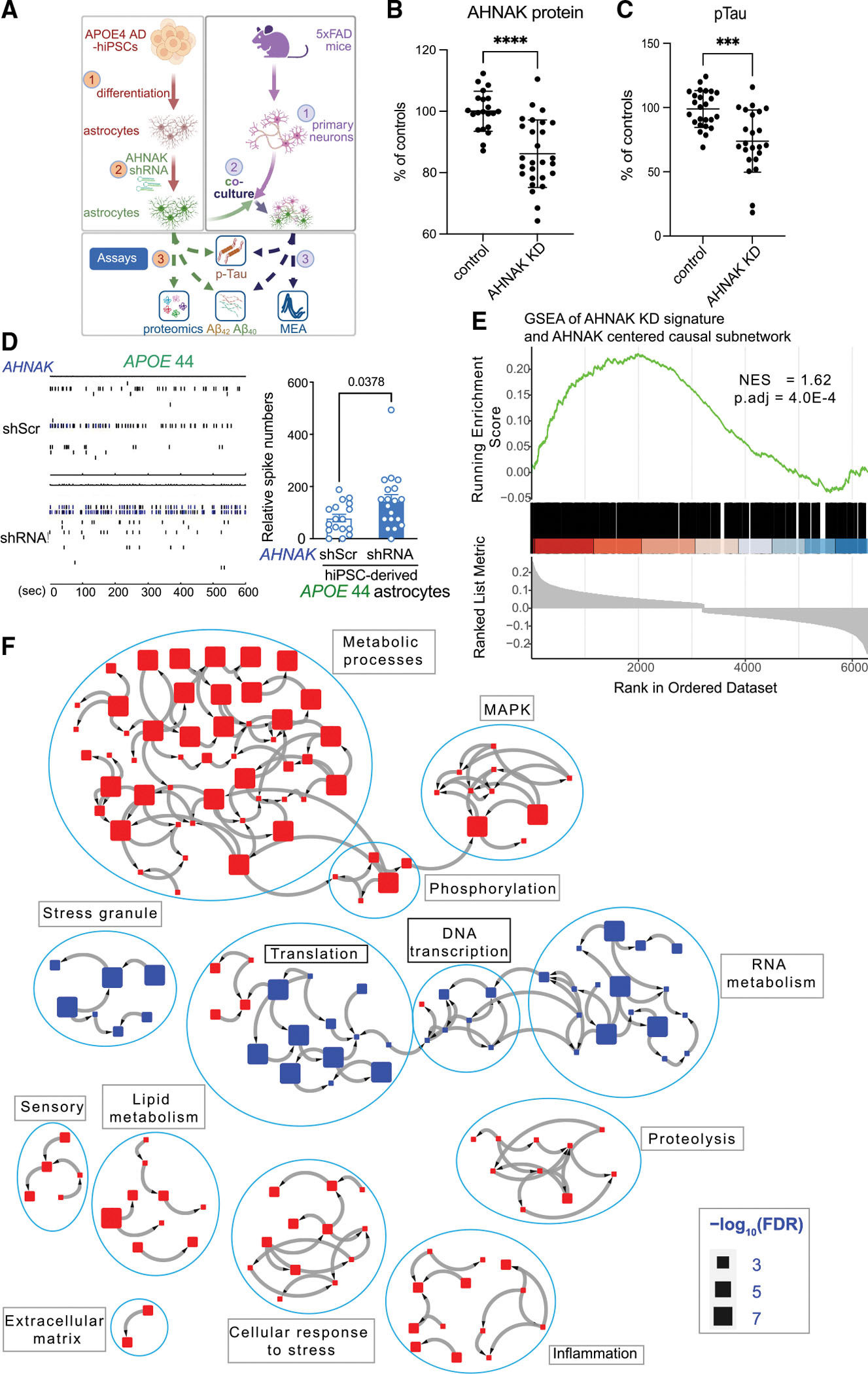
Functional validation of AHNAK (A) The overall experiment design and workflow for functional validation of AHNAK using human iPSC-derived astrocytes and their 5xFAD-mouse neuron co-cultures. (B and C) Downregulation of *AHNAK* in human iPSC-derived astrocyte culture. Levels of AHNAK (B) and pTau (C) (detected by AT270) were examined in *APOE* 44 AD human iPSC-derived astrocytes in the presence of scramble (ctrl) or AHNAK shRNA knockdown (KD) virus treatment. Results were presented as % of controls with levels in iPSC-derived astrocytes from samples treated with scramble ctrl conditions as 100%. *n* = 21–27/condition in (B) and *n* = 23–25/condition in (C). ****p < 0.0001, ***p < 0.001, **p < 0.01 with independent-samples *t* test. (D) Left: MEA representative raster plots over 10 min; right: spike analysis of 5xFAD primary cortical neurons co-cultured with lentivirus-containing *AHNAK* shRNA or scramble-shRNA (shScr)-transfected *APOE* 44 human iPSC-derived astrocytes, respectively. The raster plot indicates the neuronal activity of spiking, bursting, and synchrony. Unpaired *t* test compares the means of the numbers of spikes between the *AHNAK* shRNA and scrambled groups (*N* = 17–19 wells/condition). *p < 0.05 with independent-samples *t* test. (E) GSEA plot showing the enrichment trend of the AHNAK-regulated protein signatures in its neighborhood in the PHG proteomics MEGENA network (up to 3-walk, undirected). NES, normalized enrichment score. (F) AHNAK-regulated signaling pathway map based on GO biological process (BP) hierarchy (see STAR Methods for details). Each node denotes a GO/BP term that is significantly enriched for the *AHNAK* KD signature and is proportional to its enrichment *p* value (adjusted *p* < 0.05). The color of each node stands for the directionality of enrichment: blue and red for suppressed and activated, respectively. Each outlining circle represents a large functional cluster of GO BP terms. See also Figure S5 and Table S6, pages 2–11.

As the previous DEP analysis of patient subgroups with different APOE genotypes revealed a progressive increase in AHNAK protein expression, from APOE33-NL to APOE33-AD and then to APOE4-AD (Figure 2F), we decided to use an iPSC line derived from an *APOE* ϵ4/ϵ4 (*APOE*44) carrier. We then compared AHNAK expression levels in different human iPSC-derived astrocyte cultures and selected an *APOE* 44 AD iPSC line with high AHNAK expression. Figure 7A outlines the experimental workflow we used to validate the functional role of *AHNAK* in AD. These experiments aimed to determine whether knocking down AHNAK in astrocytes could have beneficial effects on neurons, as suggested by our network analyses highlighting significant interactions between astrocytic hub genes and neurons in the module M3 of the PHG protein co-expression network.

The *AHNAK* short hairpin RNA (shRNA) treatment downregulated its protein expression in *APOE* 44 AD astrocyte culture (83.4% of scramble control, *p* = 0.0001) (Figure 7B; Table S6, page 2). There were decreases in pTau levels in *APOE*44 AD astrocyte culture with AHNAK downregulation when compared with control (70.8% of control, *p* = 0.0001) (Figure 7C; Data S1, page 4D; Table S6, page 3). There were modest reductions in Aβ_42,_ Aβ_40,_ and APOE levels in *APOE* 44 AD astrocytes with AHNAK downregulation (8.9%, 9.4%, and 9.3% of decreases in Aβ_42_, Aβ_40_, and APOE levels, respectively) (Data S1, page 4D; Table S6, pages 4–6).

Next, we tested whether downregulation of *AHNAK* in astrocytes could exert any beneficial/protective effects on neurons when co-cultured together. The axion multi-electrode array (MEA) system was utilized to measure neuronal activities of primary cortical neurons from 5xFAD mice that were co-cultured with *APOE*44 human iPSC-derived astrocytes transfected with scramble control or *AHNAK* shRNA, respectively. At baseline, cortical neurons from 5xFAD mice (co-cultured with astrocytes treated with scramble controls) exhibited impairments in neuronal activities with limited firing recorded. However, neurons co-cultured with *AHNAK*-downregulated astrocytes showed improved electrical activities (Figure 7D). The raster plot demonstrated increased spiking, bursting, and synchrony (Figure 7D). The numbers of spikes from neurons exhibited a statistically significant enhancement in neuronal activities once co-cultured with *AHNAK*-downregulated astrocytes (1.8-fold increase; *p* = 0.0378) (Figure 7D, right; Table S6, page 7). There was a consistent reduction in pTau levels with *AHNAK* shRNA-treated astrocytes co-cultured with neurons (11.4% decrease in pTau levels, *p* = 0.002; 18% of *AHNAK* knockdown [KD], *p* < 0.0001) (Data S1, page 4E, top; Table S6, pages 8 and 9). On the other hand, there were no significant decreases in Aβ_42_ or Aβ_40_ levels seen in *AHNAK* shRNA-treated astrocytes co-cultured with neurons (Data S1, page 4E, bottom; Table S6, pages 10 and 11). Together, these findings suggested that *AHNAK* downregulation in astrocytes provided beneficial effects in promoting neuronal activities and maintaining neuronal functions, partially through reduction of pTau levels in both astrocytes and astrocyte/neuron co-cultures.

### AHNAK-regulated iPSC protein signature is enriched in its protein network neighborhood

We further investigated the underlying molecular mechanisms of AHNAK downregulation that contributed to the beneficial/protective effects. Upon downregulation of AHNAK in *APOE44* human iPSC-derived astrocytes (Figure S5A), we observed hundreds of proteins with significant expression changes (*p* < 0.05 and FC > 1.1 or < 0.91) (Figure S5B). We then performed GSEA^70^ to examine any over-representation of the AHNAK-perturbed protein signature in the AHNAK-centered network neighborhoods (STAR Methods). Indeed, the AHNAK-perturbed protein signature was enriched in the AHNAK-centered protein network neighborhood (NES = 1.6, adjusted *p* = 4.0e–4) (Figure 7E). Based on the AHNAK perturbation signature, we further constructed an AHNAK-regulated signaling pathway map involving interactions among metabolic processes, mitogen-activated protein kinase (MAPK) signaling, inflammation, and lipid metabolism, all of which have been associated with AD or cognitive functions^30,71^ (Figure 7F; STAR Methods). Taken together, these findings further validated the functional relevance of AHNAK in AD. Notably, the proteomic signature of AHNAK from the *AHNAK* KD experiments on *APOE* 44 human iPSC-derived astrocytes validated the molecular function suggested by the large-scale human brain proteomic data in AD.

## DISCUSSION

In this study, we have developed multiscale proteomic network models for AD by integrating large-scale matched proteomic and genetic data from AD-vulnerable brain regions, including the PHG and PFC. Hundreds of co-expressed protein modules were identified and characterized for their association with AD, and the top module captured the interactions among neurons, astrocytes, and microglia, highlighting dysregulated glia-neuron interactions in AD. Meanwhile, the Bayesian causal network reveals the causal relationships among thousands of proteins and enables the systematic identification of KDPs for AD. AHNAK, a top driver of AD and a hub gene in the top-ranked glia-neuron module, was experimentally validated in iPSC-based AD model systems. Downregulation of AHNAK not only rescued neurodegeneration but also induced proteomic changes that recapitulate those observed in the human brain proteomic causal network, thus demonstrating the power of the multiscale proteomic modeling of AD.

The module M3 in the MSBB PHG protein co-expression network captures the interactions among the downregulated neuronal genes, the activated astrocytic genes, and the activated microglia genes, reflecting the communications among neurons, microglia, and astrocytes.^72,73^ Among the 108 KDPs in the M3 module, 21 (19.4%) have not been studied in AD, and thus they are unexplored targets. One top target, ERBB2IP, a protein tyrosine kinase upregulated in AD, was found to contribute to poor prognosis in epidermal oncogenesis,^74^ plays a role in B cell-mediated antitumor immunity,^75^ and accelerates lysosome biogenesis and autophagy.^76^ ERBIN has been shown to interact with PSD-95 at postsynaptic membranes, potentially playing a significant role in the regulation of neuregulin signaling.^77^ ERBIN is required for myelination in regenerated axons after injury.^78^ The role of ERBIN in AD remains to be elucidated. Another downregulated KDP, OLFM3, has been shown to act as a proangiogenic factor in the tumor microenvironment.^79^ We previously highlighted OLFM3 as a potential cerebrospinal fluid (CSF) biomarker for AD.^80^

A critical step in developing AD therapeutics is target identification and prioritization.^20–22,30–32,39^ As protein networks developed from this study are more relevant to AD than transcriptomic networks and generic protein interaction networks, they offer a foundation for developing next-generation therapeutics for treating AD. In this study, we identified 580 distinct KDPs, including some known AD protein targets such as MSN and PRDX6.^20–23^ Indeed, PRDX6 was shown to play functional roles^64^ in AD, such as attenuation of ischemic oxidative damage^65^ and anti-inflammatory processes in glia.^23^ These results support the validity of our KDPs as potential targets for AD drug discovery.

In this study, we observed either no or very few DEPs between the moderate vs. the NL group, as shown in Figure 2C. Several factors may account for the lack of detectable protein changes in the brains of MCI and preclinical AD patients in this study: (1) the brains curated in the MSBB cohort did not exhibit significant molecular changes in the PHG region, as we also found no DEGs between MCI (CDR = 0.5) and nondemented (CDR = 0) subjects in the bulk RNA sequencing (RNA-seq) data from the PHG samples in our previous publication^33^; (2) MCI patients might exhibit greater molecular heterogeneity compared with AD patients; and (3) the criteria we used to identify DEPs (FDR < 0.05 and FC > 1.1 or < 1/1.1) might be too stringent, as many previous proteomic studies used a more liberal cutoff (e.g., nominal *p* value < 0.05).^20,21,29^ With the criterion of nominal *p* < 0.05, we observed 269 and 403 DEPs between MCI and nondemented (Data S1, page 6) and between impaired (CDR of 0.5, 1, and 2) and nondemented, respectively. Further research is needed to explain the absence of protein changes in the PHG region of the brain in MCI and preclinical AD patients.

In summary, this study developed multiscale proteomic regulatory network models for AD by integrating matched genetic, proteomic, clinical, and pathological data. We systematically identified co-expressed protein modules and protein network drivers involved in AD pathogenesis, highlighting a subnetwork that captures interactions among neurons, astrocytes, and microglia. These findings lay a foundation for uncovering potential molecular mechanisms underlying AD and advancing the development of next-generation therapeutics against AD.

### Limitations of the study

In this study, we analyzed the two largest AD proteomic cohorts with matched genetic and transcriptomic data, identifying shared and omics-type-specific subnetworks and drivers. While we validated AHNAK and its subnetwork, most findings require further biological and experimental validations. Even for AHNAK, *in vivo* studies are needed to clarify its role in cognition, memory, and AD pathogenesis.

## RESOURCE AVAILABILITY

### Lead contact

Further information and requests for resources and reagents should be directed to and will be fulfilled by the lead contact, Bin Zhang (bin.zhang@mssm.edu).

### Materials availability

This study did not generate unique reagents.

### Data and code availability

- The human postmortem sequencing and proteomic data are available via the AD Knowledge Portal (https://adknowledgeportal.synapse.org). The AD Knowledge Portal is a platform for accessing data, analyses, and tools generated by the Accelerating Medicines Partnership (AMP-AD) Target Discovery Program and other National Institute on Aging (NIA)-supported programs to enable open-science practices and accelerate translational learning. The data, analyses, and tools are shared early in the research cycle without a publication embargo on secondary use. Data is available for general research use according to the following requirements for data access and data attribution (https://adknowledgeportal.synapse.org/DataAccess/Instructions). The MSBB human PHG proteomics dataset is available at Synapse: syn21347564. The MSBB human PHG bulk RNA-seq dataset is available at Synapse: syn20801188. The ROSMAP bulk RNA-seq and proteomics datasets are available at Synpase: syn4164376 and Synapse: syn21261728, respectively. The BLSA human PFC proteomics dataset is available at Synapse: syn9884368. The ROSMAP snRNA-seq dataset is available at Synapse: syn52293417. The human APOE snRNA dataset is available at GEO: GSE254205. Mouse 5xFAD and WT proteomics are available at ProteomeXchange: PXD018590. DOIs for these datasets are listed in the key resources table. In addition, all of the data reported in this paper will be made available by the lead contact upon request.
- For access to content described in this manuscript see: https://www.synapse.org/Synapse:syn69761616.

## STAR★METHODS

### EXPERIMENTAL MODEL AND STUDY PARTICIPANT DETAILS

#### Human Postmortem Brain Samples

Previously, we performed bulk RNA-seq sequencing on 4 brain regions (Brodmann area (BA)10, BA22, BA36 (The parahippocampal gyrus (PHG) region), and BA44) across 364 postmortem human brains obtained from the Mount Sinai/JJ Peters VA Medical Center Brain Bank (MSBB) cohort, which holds 2,407 well-characterized brains (By querying the NIH Neurobiobank at https://neurobiobank.nih.gov/subjects/). The ages of the subjects are determined based on their Age at Death (AOD). In the current population, AOD ranged from 61 to 108 years, with an average of 84.7 years and a standard deviation of 9.7.^11^ The detailed description about the clinical and phenotypical traits for the 364 subjects was deposited on Synapse (Synapse ID: syn6101474 at https://www.synapse.org/Synapse:syn6101474). Note, for the PHG region (BA36), we conducted bulk RNA-seq assays for 215 subjects, which passed the quality control (QC) in RNA-seq.^11,33^

In this study, we further carried out proteomics profiling on the parahippocampal gyrus (PHG) of 198 postmortem normal healthy control brains, and brains with various pathology in AD from the MSBB cohort. Among the 198 brains, 185 passed the QC in proteomics (see below for QC details), which had matched bulk RNA-seq data (see Figure 1B, top panel, numbers in the brackets). The QC data was deposited on Synapse (Synpase ID: syn21347564).

For the Religious Orders Study and Memory and Rush Aging Project^90,91^ (ROSMAP) cohort, we downloaded from Synapse the proteomic data (Synapse ID: syn21261728) in the prefrontal cortex (PFC) of 400 postmortem brains with no cognitive impairment, and brains with various pathology in AD or dementia.^92–94^ We also downloaded from Synapse bulk RNA-seq data (Synapse ID: syn4164376) in the PFC from 623 postmortem brains with no cognitive impairment, and brains with various pathology in AD or dementia^92–94^ in the ROSMAP cohort. There are 208 brains that are common between the proteomics and bulk RNA-seq data (see Figure 1B, middle panel, numbers in the brackets). For the postmortem brains used in this study, the donors have an average of AOD of 89 years, ranging from 66 to 108. The individual clinical meta data for the ROSMAP cohort is available at https://www.synapse.org/Synapse:syn3191087.

For The Baltimore Longitudinal Study on Aging (BLSA) study, we downloaded from Synapse the proteomic data (Synapse ID: syn9884368) in the prefrontal cortex (PFC) of 47 postmortem brains of normal control, and brains with various pathology in AD (see Figure 1B, bottom panel, left). Among the 47 subjects, 21 have an AOD ≥ 90, whereas the remaining have an average of AOD of 78.9 years, ranging from 62 to 88.^21^ The individual clinical meta data for the BLSA cohort is available at https://www.synapse.org/Synapse:syn7417639.

#### Human Induced Pluripotent Stem Cell (iPSC) Lines

The previous published cohort human iPSC lines^95^ were utilized in this study. The caucasian background *APOE* 44 carrier of male with AD diagnosed (CDR=0.5) human iPSC (ID11) was selected^95^ for this study. The paretnal hiPSCs were derived from dermal fibroblasts collected from the UCI ADRC cohort. All hiPSC research was conducted under the oversight of the Institutional Review Board (IRB) and Embryonic Stem Cell Research Overview (ESCRO) committees at the Icahn School of Medicine at Mount Sinai (ISSMS), and Institutional Biosafety Committee at Boston University Chobanian and Avedisian School of Medicine.

#### Mouse Model

The details regarding the 5xFAD mouse model are described in Bai et al.^20^ and Oakley et al.^54^ Heterozygous 5xFAD(+/−) transgenic mice (JAX #006554), which overexpress human APP and PS1 mutations linked to familial Alzheimer’s under the Thy-1 promoter, along with littermate wild type controls, were used. Both male and female mice aged 6 months were included.

### METHOD DETAILS

#### Proteomic profiling

Proteomics profiling was conducted on 198 human postmortem brain cortex tissues in the PHG region in the MSBB cohort (Synapse ID: syn21347564). Protein extraction and quantitation, sample quality and pooling, proteomics profiling such as protein digestion and tandem-mass-tag (TMT) labeling, extensive two-dimensional liquid chromatography-tandem mass spectrometry (LC/LC-MS/MS), and qualitative identification and quantitative normalization of proteins in human brains were essentially followed the methods as described in Bai et al.^20^ and Tan et al.,^96^ We retained 185 samples that passed QC following our in-house pipeline,^11,37^ which included the initial removal of a batch of 8 samples with low quality and subsequent removal of 5 samples based on genetic concordance alignment.^11,20^ *W*e identified 12,147 distinct protein/isoforms that had a detectable expression count across all 185 samples. The normalized raw counts were log2-transformed and then corrected for the covariables including postmortem interval (PMI), race, gender, batch, and age at death via a linear model. The regression residuals were used for downstream analysis.

The proteomic data in the prefrontal cortex and subject metadata in the ROSMAP cohort were downloaded from Synapse (Synapse ID: syn32539359^28,29,38,39^). Protein entries that had missing values in more than 50% of the samples were discarded. Missing values were imputed by the function in the R package ‘impute’ with the default parameters. The expression level of the remaining proteins was further corrected by covariates of sex and age at death via a linear model. The regression residuals were used for down-stream analysis.

We downloaded the raw label-free quantification (LFQ) data of MaxQuant proteomics for the 47 PFC samples in the BLSA cohort from Synapse (Synapse ID: syn9884368). The method for preprocessing the raw data is essentially as described in the previous study.^97^ Briefly, we used the total intensity of LFQ as the abundance for the protein groups, termed proteins. We discarded proteins that were potential contaminants, decoy proteins (reverse), or lacked gene name annotation, or had missing values in more than 50% of the samples. We log2-transformed the protein expression and imputed the missing values using the function in the R package ‘impute’ with the default parameters.^98^ We applied the median centering method to normalize the protein expression.^99^ Finally, the normalized expression was further adjusted for the covariables including sex, age and PMI by the linear model, and the residuals were used for downstream analysis.

#### Bulk RNA-seq data preprocessing

The bulk RNA-seq data in the Mount Sinai Brain Bank (MSBB) cohort from patients with Alzheimer’s disease (AD) symptoms of various severities and normal healthy controls were used to identify gene expression changes in AD (Synapse ID: syn20801188^11,40,42^). Single-end RNA-seq assays were performed on total cellular RNAs extracted from the brain cortex region, parahippocampal gyrus (PHG). The raw sequence reads were aligned to the human genome hg19 with the star aligner (v2.3.0e).^100^ Then the mRNA expression was quantified and summarized at the gene level by featureCounts (v1.4.4)^101^ based on Ensembl gene model GRCh37.70. The raw read counts data were normalized using the trimmed mean of M-values normalization (TMM)^102^ method to adjust for variation in sequencing library size, transformed via voom, and further adjusted for covariates including postmortem interval (PMI), race, RNA integrity number (RIN), gender, rate of exonic reads, and batch using a linear model,^103^ where batch was treated as a random effect. The residuals from the regression model were used for downstream analysis.^42^

The bulk RNA-seq data in the ROSMAP cohort were downloaded from the AMP-AD Knowledge Portal with accession number syn3219045 and then went through read alignment, gene quantification, normalization, and covariate adjustment, as described above.^11,33^

#### Differential expressed protein (DEP) analysis

In the MSBB cohort, we used 4 AD clinical and pathological traits to capture and delineate AD-associated changes in proteomics. The 4 AD traits are: 1) the clinical dementia rating (CDR) scale for assessing dementia and cognitive status; 2) the Consortium to Establish a Registry for Alzheimer’s Disease (CERAD) score to describe a case-control status for AD; 3) the Braak score to assess progression of neurofibrillary neuropathology; and 4) the plaque mean to quantitate mean density of neuritic plaques. Modest correlation was found among these traits,^41^ thus, indicating they may function through both distinct and shared processes underpinning AD pathology.

To facilitate the identification of AD-associated proteins we typically stratified the subjects into 3 groups that represent disease severity stages from normal through moderate to high severity in AD pathology and dementia per each of the 4 traits.^18,33,40,41^ Specifically, for Braak score, subjects were classified into Low (Braak score ≤ 2), Medium (2< Braak score ≤ 4), and High (Braak score > 4) groups. With CDR, subjects were stratified into cognitive normal (Nondemented) (CDR = 0), Impaired (0 < CDR ≤ 2), and demented (CDR > 2) groups. For plaque mean density (Plaque Mean), subjects were classified into Normal (Plaque Mean = 0), Mild (0 < Plaque Mean ≤ 9), and Severe (Plaque Mean > 9) groups. For CERAD score, subjects were classified into DefiniteAD (CERAD = 2), IndefiniteAD that includes probable AD (CERAD = 3) and possible AD (CERAD = 4), and NL (CERAD = 1) (see Table S1; pages 1–2 for details).

Next, we interrogated the differential expression of proteins between any two of the three groups using the moderated t-test implemented in the limma package.^86^ False discovery rate (FDR) was then estimated by the Benjamini-Hochberg (BH)^104^ method to adjust for multiple tests. Differentially expressed proteins (DEP) were determined by BH-adjusted p value less than 0.05 and fold change in expression greater than 1.1 or smaller 1/1.1 and BH-adjusted p < 0.05 in each comparison per each trait.

A similar approach was utilized to detect DEPs in the ROSMAP and BLSA cohorts. For the ROSMAP cohort, 3 clinical traits were considered, that is, Braak score, CERAD and MMSE score. The Braak score classification was the same as in the MSBB cohort. For CERAD score, subjects were classified into DefiniteAD (CERAD = 1), IndefiniteAD that includes probable AD (CERAD = 2) and possible AD (CERAD = 3), and NL (CERAD = 4). With MMSE score, subjects were stratified into cognitive normal (Nondemented) (MMSE > 23), Impaired (17 < MMSE ≤ 23), and demented (MMSE ≤ 17) groups. For the BLSA cohort, subjects were grouped as normal control (NL), Asymptomatic AD (AsymAD), and AD.^21^ We used FDR < 0.05 for the ROSMAP and BLSA proteomics as the cutoff to select DEPs.

#### Differential expressed gene (DEG) analysis

In the MSBB cohort, we acquired the DEG lists from our previous publication. Differentially expressed genes (DEG) were determined to possess > 1.2 or <1/1.2 fold change in expression and BH-adjusted p < 0.05.^33^ For the ROSMAP cohort, we performed the DEG analysis across 3 clinical traits (Braak score, CERAD and MMSE) using the same method as for the ROSMAP proteomics. DEGs were identified to have an FDR < 0.05.

#### Composite differential expression (DE) analysis

To facilitate comparisons of differentially expressed (DE) signatures across different omics/cohorts, we defined a composite DEP/DEG to summarize and aggregate DEP/DEG signatures across different AD traits. For the MSBB proteomics, we obtained the DEP signatures between the most severe and least severe groups across each of the four clinical traits, that is, Demented vs. Non-demented for CDR, DefiniteAD vs. NL for CERAD, High vs. Low for Braak score, and Severe vs. Normal for plaque mean. The DEP signature was further classified into up- or down-regulated ones. A protein more frequently up-regulated than down-regulated was defined as a consensus up-regulated DEP. Conversely, a protein more frequently down-regulated than up-regulated was defined as a consensus down-regulated DEP. For each consensus up-regulated or down-regulated DEP, the smallest p-value corresponding to the direction of regulation was used to represent the significance level of differential expression. Consensus DEGs were identified in the same way. This process was also applied to the DEGs and DEPs in the ROSMAP cohort.

#### Analysis of concordance between DEPs and DEGs

The matched transcriptomic and proteomic data in both ROSMAP and MSBB provides us a valuable opportunity to compare differential protein and gene expression in the same brain region from the same set of donors. To simplify the work, we focused on the consensus DEGs and DEPs. Based on the definitions of consensus DEGs and DEPs, the genome or proteome in each cohort was classified into three groups including up-regulated DEGs (or up-regulated DEPs), down-regulated DEGs (or down-regulated DEPs) and non-DEGs (non-DEPs). Then, we determined the proportion for each of the 9 possible combinations of the DEP and DEG groups, and the statistical significance for each combination by the hypergeometric test. We intended to find out if the changes in mRNA and protein expression for each gene were in the same direction or not with respect to AD pathology.

For sex-wise DEP analysis, a special treatment was made to address the imbalance in sample sizes of male and female AD groups. There are 31, 49, 29, and 27 subjects in the female-NL, female-DefiniteAD, male-NL, and male-DefiniteAD groups, respectively. Since there are approximately twice as many subjects in the female-DefiniteAD group as in the male-DefiniteAD group, we down-sampled the female-DefiniteAD group to match the number of subjects (27) in the male-DefiniteAD group. Similarly, we down-sampled the female-NL group to match the number of subjects (29) in the male-NL group. Then, we performed the DEP analysis on the down-sampled female-DefiniteAD group vs. the female-NL group. We repeated the down-sampling process and the DEP analysis for 1,000 times. The DEPs with an occurrence frequency greater than 80% were included in the final DEP signature between female-DefiniteAD vs. female-NL.

#### Correlation analysis

The Spearman’s correlation between protein expression level and individual AD clinic traits except for plaque mean was calculated, whereas Pearson’s correlation was obtained for plaque mean. The Pearson method was used to calculate correlation between the expression of individual genes (mRNAs) and proteins.

#### Co-expression network analysis

Gene co-expression networks were identified using Multiscale Embedded Gene co-Expression Network Analysis (MEGENA).^57,105,106^ Briefly, MEGENA selects gene pairs with significant correlations (FDR < 0.05) and then embeds them onto a 3-dimensional topological sphere.^107^ The resulting co-expression network belongs to a class of geometrical networks called “planar filtered networks (PFNs)”, which can be drawn on the sphere’s surface without any link intersections.^107^ PFNs then go through unsupervised clustering to identify network clusters (i.e., gene modules) at various resolutions.^57,105,106^ The resulting gene modules are organized in a hierarchy. The hierarchy represents the multiscale organization of gene modules with different degrees of compactness. It captures a series of higher-order relationships (i.e., parent) modules possessing children modules residing within these parent modules. MEGENA recognizes more compact children modules within the parent modules.^57^

Furthermore, candidate key drivers of gene modules are identified, employing statistically significant hubs with hub p-value < 0.05.^57^ Gene modules are then annotated by the enriched MSigDB^108,109^ signatures (version 6.1) and associated with outcomes through the previously identified gene signatures’ enrichment test. Detection of hub genes of modules was achieved via the internal function in the MEGENA package.^57^ Essentially, hubs are those nodes with top connections with other nodes in the same individual modules.^87,110^ Key driver analysis (KDA) on MEGENA modules was performed by the R package KDA (version 0.02) following the method described in.^88,111^

#### MEGENA module preservation analysis

Module preservation analysis was essentially the same approach as in weighted gene correlation network analysis (WGCNA).^25^ Briefly, the network-based preservation score (Zsummary.pres) was calculated using the modulePreservation function from the WGCNA package. We define a module as preserved if Zsummary.pres > 6 otherwise not preserved.

#### Identification of the PHG-specific protein modules

We defined the MSBB PHG-specific protein modules as those which are not conserved in any of three MEGENA based co-expression networks including the MSBB PHG gene network, the ROSMAP PFC gene network, and the ROSMAP PFC protein network. A module m_phg-p_ in the MSBB PHG protein network is conserved in an independent network *N* if it satisfies the following three criteria: 1) m_phg-p_ is preserved (preservation score > 6, which is the average between the high and medium preservation scores^25^) in the molecular dataset that was used to construct *N*; 2) m_phg-p_ shares at least 20% of its member proteins with at least one module in *N* and at least one such overlap is significant (i.e., multiple testing corrected p-value < 0.05 by the hyper-geometric test). In this way, we identified protein modules specific not only to the PHG region but also to proteomics. We then annotated the PHG-specific protein modules by enrichment test for the known glia gene signatures such as the disease-associated astrocytes (DAA), disease-associated microglia (DAM) and homeostatic microglia (HM) in human,^60^ and the recently reported signatures for microglia states in human.^61^

#### Bayesian causal network analysis

Bayesian causal network was constructed by integrating genome-wide gene expression, single-nucleotide polymorphism (SNP) genotype, and known transcription factor (TF)-target relationships. Briefly, we computed expression quantitative trait loci (eQTLs) from the present bulk proteomics data and matched whole genome sequence (WGS) genotype using MatrixEQTL.^112^ Significant eQTLs were defined as those with false discovery rate less than 0.05. We then employed a formal statistical causal inference test (CIT)^113^ to infer the causal probability between gene/protein pairs associated with the same eQTL. The causal relationships inferred were used, together with transcription factor (TF)-target relationships from the ENCODE project, as structural priors for building a causal gene regulatory network from the gene expression data through a Monte Carlo Markov Chain (MCMC) simulation-based procedure.^17,40,89^ We followed a network averaging strategy in which 1,000 networks were generated from the MCMC procedure starting with different random structure, and links that shared by more than 30% of the networks were used to define a final consensus network structure. To ensure the consensus network is a directed acyclic graph, an iterative de-loop procedure was conducted, removing the most-weakly supported link of all links involved in any loop. Following the method by Zhang et al.,^10^ we performed key driver analysis (KDA) on the consensus Bayesian network to identify key hub genes, which regulated many downstream nodes.

#### Differentially expressed protein (DEP) analysis on 5xFAD mice

Cortical brain tissue samples of 5xFAD female/male mice of 6 months were used for protein extraction and proteomics profiling following the method described in Bai et al.^20^. The raw protein expression was normalized by log2-transformation, and further adjusted for sex by the linear model, and the residuals were used for downstream analysis. Differential expression analysis was carried out by the moderated t-test implemented in the limma package^86^ to detect DEPs in 5xFAD vs. wildtype mice.

#### Quantitative capillary western (Wes) assays^114^

Protein abundance was measured in the perirhinal cortex (PHG) from DefiniteAD (N=9) and NL subjects (N=10). Optimal protein lysis solutions were evaluated in preliminary studies. Aliquots (25 mg) of grey matter from the snap-frozen the perirhinal cortex were pulverized in a liquid nitrogen-cooled mortar and homogenized in Tris/EDTA lysis buffer (20mM Tris pH 8.0, 0.2 mM EDTA, 150 mM NaCl, 3% NP40, 1% sodium deoxycholate, 0.1% SDS, 1mM PMSF and protease inhibitor cocktail (Sigma). The total protein amount was determined with a Qubit protein assay (Invitrogen). A working concentration (2 mg/ml) for each sample was prepared. Capillary Western assays were performed using the ProteinSimple Wes System (BioTechne). The linearity of each protein’s detection in whole-tissue extracts was determined in preliminary studies (from 1.2 to 0.02 mg/ml). A linear regression fit was used to determine the linear dynamic ranges of analyzed proteins. Each sample (0.4 mg/ml) was diluted with 0.1x Sample Buffer, and Fluorescent Master Mix was added. The samples were denatured at 95°C for 5 min. After denaturation, the prepared samples in duplicate, blocking reagent, primary antibody dilutions were 1:50 for MSN (ab151542), PRDX6 (ab133348, both from Abcam), RPH3A (NBP2–95144, Novus); 1:20 for VIM (NBP1–85814, Novus), NRN1 (PA5–100209, Thermo), and 1:10 for OLFM3 (PA5–49313,Thermo), HRP-conjugated secondary antibodies, and chemiluminescent substrate were dispensed into designated wells in an assay plate. A biotinylated ladder provided molecular weight standards for each assay. The separation electrophoresis and immunodetection steps took place in the fully automated Wes capillary system.

Assay analyses were performed using Compass for Simple Western^™^ (ver. 3.1.7). Areas under detected peaks in each Wes assay-module were normalized relative to the ‘standard-calibrator’ (a mix of small aliquots from all samples) to account for assay-to-assay variability. According to the manual, the sample gels were stained with KryptonTM fluorescent protein stain (ThermoFisher) to control for equal loading. Detection of protein bands was performed by image scan and analyzed using a LI-COR Odyssey Imaging System (ver. 3.0.3, LI-COR Biosciences). Statistical tests were performed with SPSS version 24.0 for Windows (IBM).

#### ELISA

Levels of human AHNAK protein in triplicate were measured by sandwich ELISA (LSBio, WA) according to the manufacturer’s manual. Linearity of the assay was confirmed by the AHNAK protein standards. Fresh frozen tissue homogenates were prepared by ultrasonication in PBS. Cellular debris were removed by centrifugation. HRP enzymatic products were measured on a microplate reader – SpectraMax 190 (Molecular Devices, CA).

#### Human iPSC-derived astrocyte and neuron/astrocyte co-culture with gene perturbation

The iPSCs derived from an *APOE* 44 AD subject^95^ were differentiated into neural progenitor cells (NPCs) by dual SMAD inhibition followed by neural rosette selection, and forebrain-specific patterning by 20ng/ml FGF2 exposure as described.^115,116^ These NPCs were differentiated to a homogeneous population of astrocytes^116,117^ before subjected to viral transfection and further analysis. The cultured *APOE* 44 iPSC-derived astrocytes were transfected with lentivirus-containing *AHNAK* shRNA (Multiplicity of Infection (MOI) 20) or scramble controls for additional 10–14 days before subjected for western blot, ELISA (enzyme-linked immunosorbent assay) or proteomic analysis. Alternatively, the transfected *APOE* 44 iPSC-derived astrocytes were co-cultured with mouse primary cortical neurons obtained from 17-day old embryos of 5×FAD mice for 2–3 weeks before subjected for western blot, ELISA (enzyme-linked immunosorbent assay) or multi-electrode array (MEA)^118,119^ analysis.

#### Multi-electrode array (MEA)

The neuronal activities were measured by MEA in co-cultures of primary cortical neurons from 5xFAD mice^54^ with *APOE* 44 human iPSC-derived astrocytes in the presence of scramble *versus AHNAK* shRNA virus, respectively. *AHNAK* shRNA or scramble-shRNA (shScr)-containing virus transfected astrocytes were seeded at 17,000 cells/well in a Matrigel-coated 48-well MEA plate (Axion Biosystems, M768-tMEA-48W). On day 5, embryonic day 17 primary cortical neurons derived from 5×FAD mice were prepared and seeded at 70,000 cells/well on the monolayers of astrocyte culture. The outer space of each well in the plate was filled up with autoclaved/deionized water to minimize the evaporation of marginal wells (“edge effect”) during long-term culture. Half of the neuronal medium was exchanged with the fresh medium until the end of the MEA recording. On the recording day, the plate was loaded into the Axion Maestro MEA reader (**Axion Biosystems**). The recording was performed via AxiS 2.4 for 10 mins. Quantitative analysis of the recording was exported as a Microsoft excel sheet. The plate map and raster plot were generated using the Neural Metric Tool (Axion BioSystems). The numbers of spikes were analyzed using GraphPad Prism 9 (GraphPad Software, Inc).

#### Antibodies and reagents

The anti-pTau AT270 (mouse monoclonal Ab, ThermoFisher, RRID: MN1050; 1:1000), anti-β-actin (mouse monoclonal Ab, Santa Cruz Biotechnology, RRID:AB_476697; 1:10000), as well as anti-mouse and rabbit HRP (ThermoFisher, RRID:AB_2556542 and 2540618; 1:10000) were purchased for western blot analysis. The human AHNAK ELISA kits (Aviva), human Aβ_42_ and Aβ_40_ ELISA kits (ThermoFisher) as well as human APOE ELISA kits (Abcam) were purchased and followed the manufactures’ instructions for detection. Lentivirus-containing AHNAK shRNA and scramble controls were generated and obtained from ABM Inc.

#### Protein extraction, digestion and TMT labeling for the human iPSC-derived astrocytes

The astrocyte cell pellets were lysed by sonication (30% power, 30 s) in 100 μL of fresh lysis buffer [50 mM HEPES, pH 8.5, 8 M urea, 0.5% sodium deoxycholate and 1 × phosphatase inhibitor cocktail (PhosSTOP, Sigma-Aldrich)]. The protein concentration was estimated by Coomassie-stained short SDS gel as previously described.^120^ Each protein sample was digested and TMT labeled using our optimized protocol.^121,122^ Briefly 100 μg of protein from each sample was digested in the lysis buffer with Lys-C (Wako, 1:100 w/w) at room temperature (RT) for 3 h, then lower urea concentration to 2 M by adding 50 mM HEPES (pH 8.5). Further digestion was performed with trypsin (Promega, 1:50 w/w) at 21 °C overnight. The digested peptides were reduced by dithiothreitol (DTT, 1 mM) for 30 min, alkylated by iodoacetamide (IAA, 10 mM) at dark for 30 min, and finally quenched with DTT (30 mM) for 30 min. The peptides were acidified by the addition of 1% trifluoroacetic acid (TFA), then centrifuged at 21,000 × g for 10 min to remove pellets. Each supernatant was desalted with an Ultra-Micro SpinColumn (Harvard apparatus) using the standard protocol. The desalted peptide eluate was dried by speedvac. All the samples were dissolved in 50 mM HEPES, pH 8.5 (~2 μg/μL), reacted with 150 μg of TMTpro reagents (The initial 8 channels) for 30 min, then quenched with 0.5% hydroxylamine for 15 min. The labeled samples were pooled equally, and desalted again before LC/LC–MS/MS analysis.

#### Extensive LC/LC-MS/MS analysis for the human iPSC-derived astrocytes

The pooled TMT labeled peptides were fractionated by an offline basic pH RPLC with XBridge C18 columns (3.5 μm particle size, 2.1 mm × 15 cm, Waters; buffer A: 10 mM ammonium formate, pH 8.0; buffer B: 90% AcN, 10 mM ammonium formate, pH 8.0). The peptides were eluted in a 160 min gradient of 15–50% buffer B. Each fraction was collected with 0.5 min, and all the fractions were concatenated to 40 fractions as previously described.^122^ The concatenated fractions were dried and then dissolved in 5% formic acid for acidic pH LC-MS/MS analysis using our optimized protocol.^121^ Briefly, samples were analyzed with C18 column (75 μm × 20 cm with 1.9 μm C18 resin, heated at 65 °C to reduce back pressure) coupled with a Q Exactive HF Orbitrap MS (Thermo Fisher Scientific). A 60 min LC gradient of 15–45% buffer B (buffer A: 0.2% formic acid, 5% DMSO; buffer B: buffer A plus 65% AcN) was used to separate peptides. MS settings included MS1 scans (60,000 resolution, 460–1600 *m*/*z* scan range, 1 × 10^6^ AGC, and 50 ms maximal ion time) and 20 data-dependent MS2 scans (60,000 resolution, starting from 120 *m*/*z*, 1 × 10^5^ AGC, 120 ms maximal ion time, 1.0 *m*/*z* isolation window with 0.2 *m*/*z* offset, HCD, 32% specified normalized collision energy (NCE), and 10 s dynamic exclusion).

#### Identification and quantification of proteins by JUMP software for the human iPSC-derived astrocytes

The protein identification and quantification were performed by the JUMP search engine.^123^ The human protein database was generated by combining downloaded Swiss-Prot, TrEMBL, and UCSC databases and removing redundancy (83,955 entries). The false discovery rate (FDR) was estimated using target-decoy database.^124^ The database search was performed using the following major search parameters: 15 ppm mass tolerance for precursor and product ions, fully tryptic restriction, two maximal missed cleavages, static modification for TMT tags (+304.20715 on Lys and N-termini) and carbamidomethylation (+57.02146 on Cys), dynamic modification for oxidation (+15.99491 on Met). The resulting PSMs were filtered by mass accuracy and then grouped by precursor ion charge state followed by the cutoffs of JUMP-based matching scores (Jscore and ΔJn) to reduce FDR below 1% for proteins. When the same peptide was derived from numerous homologous proteins, the peptide was matched to the protein with the top PSM number, according to the rule of parsimony. Finally, the protein quantification was performed using the TMT reporter ion intensities as previously described.^125^

#### DEP and Gene Set Enrichment Analysis (GSEA) analysis of the human iPSC-derived *AHNAK*-knockdown (KD) astrocytes

The expression of the above-quantified proteins were log2-transformed and normalized by median centering.^99^ The DEP analysis was performed on individual proteins in *AHNAK* KD vs. mock control by the moderated t-test implemented in the limma package.^86,126^ This DEP signature was rank-ordered according to its fold-change in *AHNAK* KD vs. mock control in descending order. The Gene Set Enrichment Analysis (GSEA) was carried out to examine any over-representation of the sorted AHNAK-KD signature in the AHNAK-centered network neighborhoods (up to 3-layers in the MSBB MEGENA proteomics network) using the R package clusterProfiler.^70^

#### Construction of AHNAK signal map

The AHNAK signaling map was generated by extracting the gene ontology (GO) hierarchy network of the biological process terms showing significant enrichment for the AHNAK-KD DEP signature by GSEA^70^ (q value < 0.05). Specifically, we retrieved the human GO annotation data from the R/Bioconductor packages org.Hs.eg.db^127^ and GO.db^128^ and then conducted the overlap analysis between GO terms and KD signature using the hypergeometric test. Lastly, the AHNAK signaling map was visualized in Cytoscape (version 3.7.2).

#### Cross-Cell-type Interactions

To investigate the existence of the cross-celltype interactions in human single cell RNA data, we generated pseudobulk expression data from a published ROSMAP single-nucleus RNA-sequencing (snRNA-seq) dataset.^59^ Nuclei from the same donor were merged by celltype to create celltype-specific pseudobulk data, which was subsequently normalized by trimmed mean of M values (TMM)^129^ as we previously did.^33^ This dataset contains 392 prefrontal cortex brain samples from 144, 102 and 146 subjects with Alzheimer’s disease (AD), mild cognitive impairment (MCI) and no cognitive impairment (NCI), respectively. The subjects were classified based on the final consensus cognitive diagnosis score (cogdx = 4, 2, and 1 for AD, MCI and NCI, respectively). To facilitate the calculation of cross-celltype interactions, we added celltype annotations to each gene symbol, treating genes with the same name in different cell-types as distinct genes. We employed the Pearson correlation method to calculate gene-gene correlation, and adjusted p-values for multiple testing using the Benjamini-Hochberg (BH) method. We define significant interactions as the gene pairs that have an absolute value of correlation coefficient (rho) > 0.2 and p. adj < 0.05.

#### *AHNAK* cell-type-specific expression analysis

To determine the cell-type specificity of *AHNAK* expression, we downloaded a published single-nucleus RNA-sequencing (snRNA-seq) dataset (GEO: GSE254205),^81^ which was derived from fresh-frozen frontal cortex tissue from postmortem human brains of AD and sex-matched control individuals with various *APOE* genotypes.^81^

We retrieved the raw count data using the scanpy package^84^ (version 1.10.1), and then re-processed the data using the sctransform-based pipeline^130^ from the R package Seurat.^131^ For the initial quality check (QC), we excluded cells that have a total count of unique features (genes) over 7,500 or less than 200. In addition, we excluded cells that had a mitochondria-derived count > 5%. After QC filtering, we retained a total number of 109,791 unique nuclei. We pre-clustered all the nuclei and identified 14 distinct pre-clusters. Using known gene markers, we profiled and annotated all the major human brain cell types: excitatory neurons (Ex), inhibitory neurons (In), astrocytes (Ast), microglia (Mic), oligodendrocytes (Olig), oligodendrocyte progenitor cells (Opc), and endothelial cells (Endo).

#### PubMed literature mining

The function EUtilsSummary (type = “esearch”, db=“pubmed”, datetype=‘pdat’) from the R package RISmed (version 2.3.0) was used to query and download content from Pubmed. The query words were built as “KDP and Alzheimer’s disease” where the word KDP was replaced by the protein gene symbol of a specific KDP. For example, the query of “MSN and Alzheimer’s disease” would pull out all the papers that have the occurrence of MSN and Alzheimer’s disease.

### QUANTIFICATION AND STATISTICAL ANALYSIS

Levels of pTau were normalized to β-actin levels and expressed as percentage of control. Absolute AHNAK, Aβ_42_, Aβ_40_ and APOE concentrations were quantitatively determined by ELISA and expressed as percentage of control. The ANOVA with post hoc tests were used to determine group differences for multiple comparisons. Equality of variance was checked for all statistical comparisons. When independent-samples *t* tests were used and equality of variances of compared groups were not the same, the Mann-Whitney tests were applied. All statistical analysis was performed using Prism 9.0 or above.

For bioinformatics analysis, all of the statistical significance levels reported in this study were adjusted for multiple testing (BH) unless otherwise stated. For brevity, all FDRs less than 2.2e–100 were indicated with <2.2e–16. Statistical analysis was performed using either R (version 3.5.3 or above).

## Supplementary Material

7

6

4

2

5

3

1

8

SUPPLEMENTAL INFORMATION

Supplemental information can be found online at https://doi.org/10.1016/j.cell.2025.08.038.

## Figures and Tables

**KEY RESOURCES TABLE:** 

REAGENT or RESOURCE	SOURCE	IDENTIFIER
Antibodies		
Rabbit anti-MSN	Abcam	Cat#ab151542; RRID:AB_2893185
Rabbit anti-PRDX6	Abcam	Cat# ab133348; RRID:AB_11155931
Rabbit anti-RPH3A	Novus	Cat# NBP2-95144; RRID:AB_3464884
Rabbit anti-VIM	Novus	Cat# NBP1-85814; RRID:AB_11020836
Rabbit anti-NRN1	Thermo	Cat# PA5-100209; RRID:AB_2815739
Rabbit anti-OLFM3	Thermo	Cat# PA5-49313; RRID:AB_2634767
Mouse monoclonal anti-pTau AT270	ThermoFisher	Cat# RRID: MN 1050
Mouse monoclonal anti-β-actin	Santa Cruz Biotechnology	Cat# RRID: AB_476697
Secondary anti-mouse	ThermoFisher	Cat# RRID: AB_2556542
Secondary anti-rabbit	ThermoFisher	Cat# RRID: AB_2540618
Goat anti-rabbit secondary HRP conjugate	Protein Simple	Cat# 042-206; RRID:AB_2860577
Anti-Mouse Detection Module for Jess/Wes (includes goat anti-mouse secondary HRP conjugate)	Protein Simple	Cat# DM-002; RRID:AB_3095298
Biological samples		
Human postmortem brain samples	Mount Sinai/JJ Peters VA Medical Center Brain Bank (MSBB)	https://icahn.mssm.edu/research/nihbrain-tissue-repository
Human postmortem brain samples	The Rush Alzheimer’s Disease Center (RADC)	https://www.radc.rush.edu/
Human postmortem brain samples	The Baltimore Longitudinal Study of Aging (BLSA)	https://www.blsa.nih.gov/
Critical commercial assays		
The human AHNAK ELISA kits	Aviva (https://www.avivasysbio.com/)	Cat# OKCD00575
The human Aβ_40_ ELISA kits	ThermoFisher	Cat# KHB3481
The human Aβ_42_ ELISA kits	ThermoFisher	Cat# KHB3441
The Matrigel-coated 48-well MEA plate	Axion Biosystems	Cat# M768-tMEA-48W
The human APOE ELISA kits	Abcam	Cat# ab108813
Deposited data		
The MSBB human PHG proteomics dataset	This paper	https://www.synapse.org/#!Synapse:syn21347564
The ROSMAP human PFC proteomics dataset	Johnson et al.^30^	https://www.synapse.org/#!Synapse:syn21261728
The BLSA human PFC proteomics dataset	Seyfried et al.^21^	https://www.synapse.org/#!Synapse:syn9884368
The ROSMAP human PFC bulk RNA-seq dataset	Bennett et al.^81^	https://www.synapse.org/Synapse:syn4164376
The MSBB human PHG bulk RNA-seq dataset	Wang et al.^11,33^	https://www.synapse.org/#!Synapse:syn20801188
The ROSMAP snRNA-seq dataset	Mathys et al.^59^	https://www.synapse.org/Synapse:syn52293417
The human APOE snRNA dataset	Haney et al.^81^	GEO: GSE254205
Mouse 5XFAD and WT proteomics	Bai et al.^20^	(http://www.proteomexchange.org) with accession number of PXD018590
Experimental models: Organisms/strains		
Mouse 5XFAD model	The Jackson Laboratory, ME, USA	https://www.jax.org/strain/008730
Oligonucleotides		
AHNAK shRNA target sequences pooll: GCTCCTCACCTGGAAGTAAATCCCTCCTC	ABM Inc.	https://www.abmgood.com
AHNAK shRNA target sequences pool2: GGGCAGAAGGTGAGATTAAAGTTCCTGAT	ABM Inc.	https://www.abmgood.com
AHNAK shRNA target sequences pool3: ACTGAAAGGCTCCAAATTTAAGATGCCTA	ABM Inc.	https://www.abmgood.com
AHNAK shRNA target sequences pool4: GAAGTGGATGTCAAACTTAAAAAGCCAGA	ABM Inc.	https://www.abmgood.com
Software and algorithms		
R (version 4.0.3 or above)	R Core Team	https://www.r-project.org/
rstudio (version2022.12.0 Build 353)	R Core Team	https://posit.co/download/rstudio-desktop/
MEGENA (1.3.7)	Song et al.^57^	https://github.com/songw01/MEGENA
ComplexHeatmap	Gu et al.^82^	RRID: SCR_017270
Hierarchy Sunburst Plot	https://github.com/mw201608/sunburst.shiny	https://network.shinyapps.io/sunburst/
Seurat	Stuart et al.^83^	RRID: SCR_016341
scanpy	Wolf et al.^84^	RRID: SCR_018139
Cytoscape (version 3.7.3)	The Cytoscape Consortium	https://cytoscape.org/
Gephi (version 0.9.7)	The Gephi Consortium	https://gephi.org/
SuperExactTest (1.1.0)	Wang et al.^85^	https://github.com/mw201608/SuperExactTest
Limma (3.54.2)	Ritchie et al.^86^	https://bioconductor.org/packages/release/bioc/html/limma.html
clusterProfiler (4.6.2)	Yu et al.^70^	https://yulab-smu.top/biomedical-knowledge-mining-book/universal-api.html
RISmed (version 2.3.0)	CRAN	https://CRAN.R-project.org/package=RISmed
NetWeaver (0.0.9)	CRAN	https://github.com/mw201608/NetWeaver
Prism7 or above	GraphPad Software, Inc	https://www.GraphPad.com
groupCor (R package version1.0)	This paper	https://github.com/wange230/proteomics_networks
The weighted gene correlation network analysis (WGCNA)	Langfelder et al.^87^	https://CRAN.R-project.org/package=WGCNA
Module conservation for MEGENA	This paper	https://github.com/wange230/proteomics_networks
Signal map	This paper	https://github.com/wange230/proteomics_networks
RIMBANET	Zhu et al.^88,89^	https://icahn.mssm.edu/research/genomics/about/resources
BioRender	BioRender	www.biorender.com
Adobe illustrator (version 27.5)	Adobe Create Cloud	https://creativecloud.adobe.com/
Code used in this paper	This paper	https://github.com/wange230/proteomics_networks

## Highlights

- A glia-neuron protein co-expression subnetwork is most strongly associated with AD
- A protein causal network is constructed by integrating genetic and proteomic data
- Key driver proteins of AD progression are identified
- Downregulation of the astrocytic driver AHNAK reduces pTau and Aβ levels
