# Supplementary material for: Multiscale proteomic modeling reveals protein networks driving Alzheimer’s disease pathogenesis
Source: Cell. Author manuscript; Available in PMC 2026 Jan 28. (PMC12851831; doi:10.1016/j.cell.2025.08.038)
Supplement: 1 [file NIHMS2129767-supplement-1.pdf]

Cell, Volume *188*

Supplemental information

Multiscale proteomic modeling reveals protein networks driving Alzheimer's disease pathogenesis

Erming Wang, Kaiwen Yu, Jiqing Cao, Minghui Wang, Pavel Katsel, Won-min Song, Zhen Wang, Yuxin Li, Xusheng Wang, Qian Wang, Peng Xu, Gefei Yu, Li Zhu, Jia Geng, Parnian Habibi, Lu Qian, Tony Tuck, Aiqun Li, Julia TCW, Panos Roussos, Kristen J. Brennand, Vahram Haroutunian, Erik C.B. Johnson, Nicholas T. Seyfried, Allan I. Levey, David A. Bennett, Junmin Peng, Dongming Cai, and Bin Zhang

Data S1

TABLE OF CONTENTS

Data S1, page 1. Summary 1 of clinical traits-associated proteins in the MSBB PHG proteomics by correlation analysis, related to Figure 2.

Data S1, page 2. The enrichment of the differentially expressed proteins from the MSBB cohort for the human celltype and the AD GWAS genetic signatures, related to Figure 2.

Data S1, page 3. Summary of the numbers of papers where individual KDPs and Alzheimer's disease co-occur, related to Figure 6.

Data S1, page 4. Experimental and functional validation of key driver proteins (KDP) for the MSBB cohort, related to Figure 7.

Data S1, page 5. Astrocyte-specific expression of the AHNAK gene in the human brain PFC, related to Figure 7.

Data S1, page 6. Volcano plot showing proteins that were differentially expressed in the PHG region in mild cognitive impairment (MCI, CDR = 0.5) vs. Nondemented (CDR = 0) subjects, related to Figure 2.

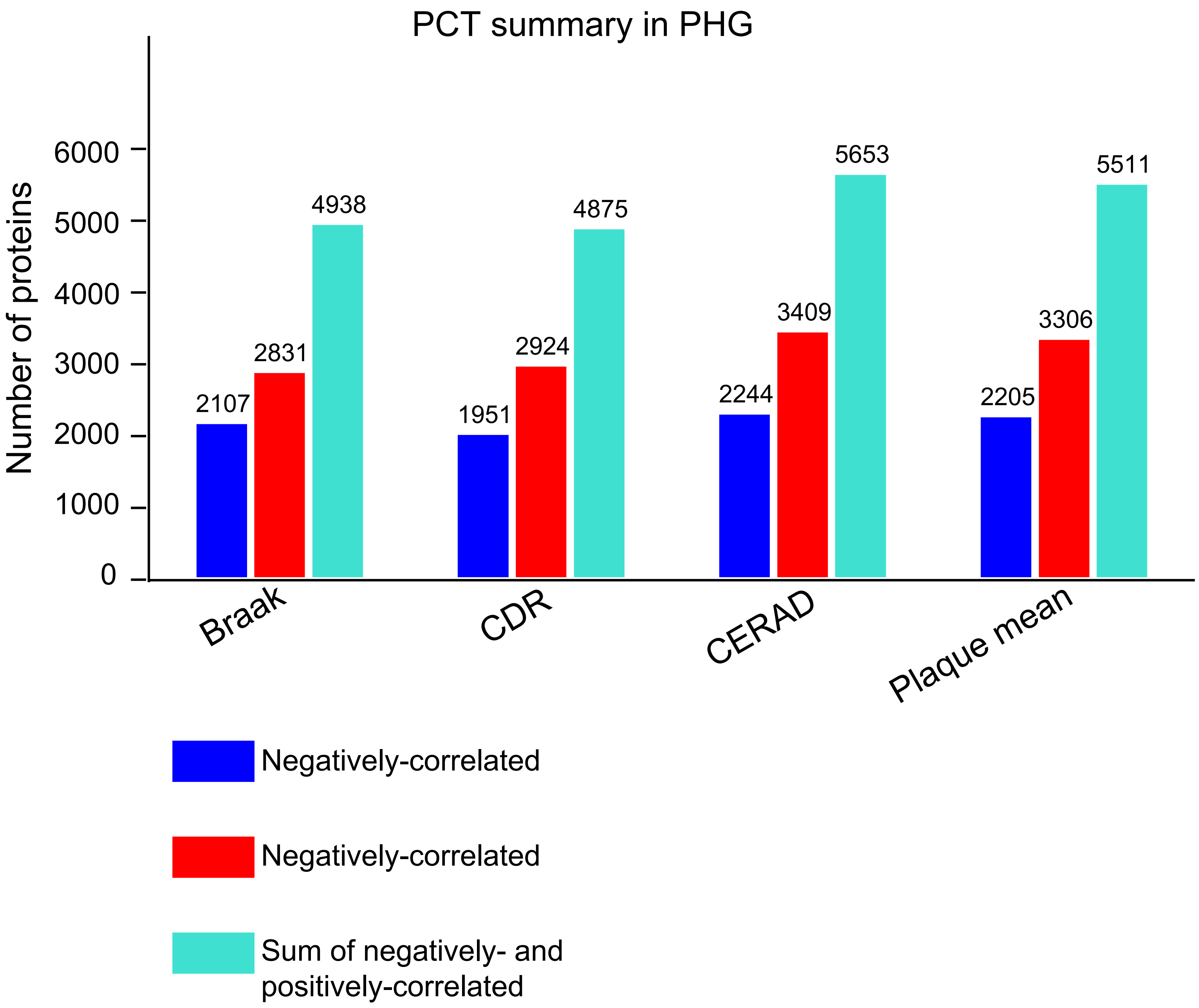
PCT summary in PHG
Number of proteins
6000
5000
4000
3000
2000
1000
0
2107
2831
4938
1951
2924
4875
2244
3409
5653
2205
3306
5511
Braak
CDR
CERAD
Plaque mean
Negatively-correlated
Negatively-correlated
Sum of negatively- and positively-correlated

Data S1, page 1. Summary 1 of clinical traits-associated proteins in the MSBB PHG proteomics by correlation analysis

About 5,000 AD-associated proteins termed as protein correlated with traits (PCT) were identified across 4 clinical traits. More positively than negatively associated PCTs were found.

A

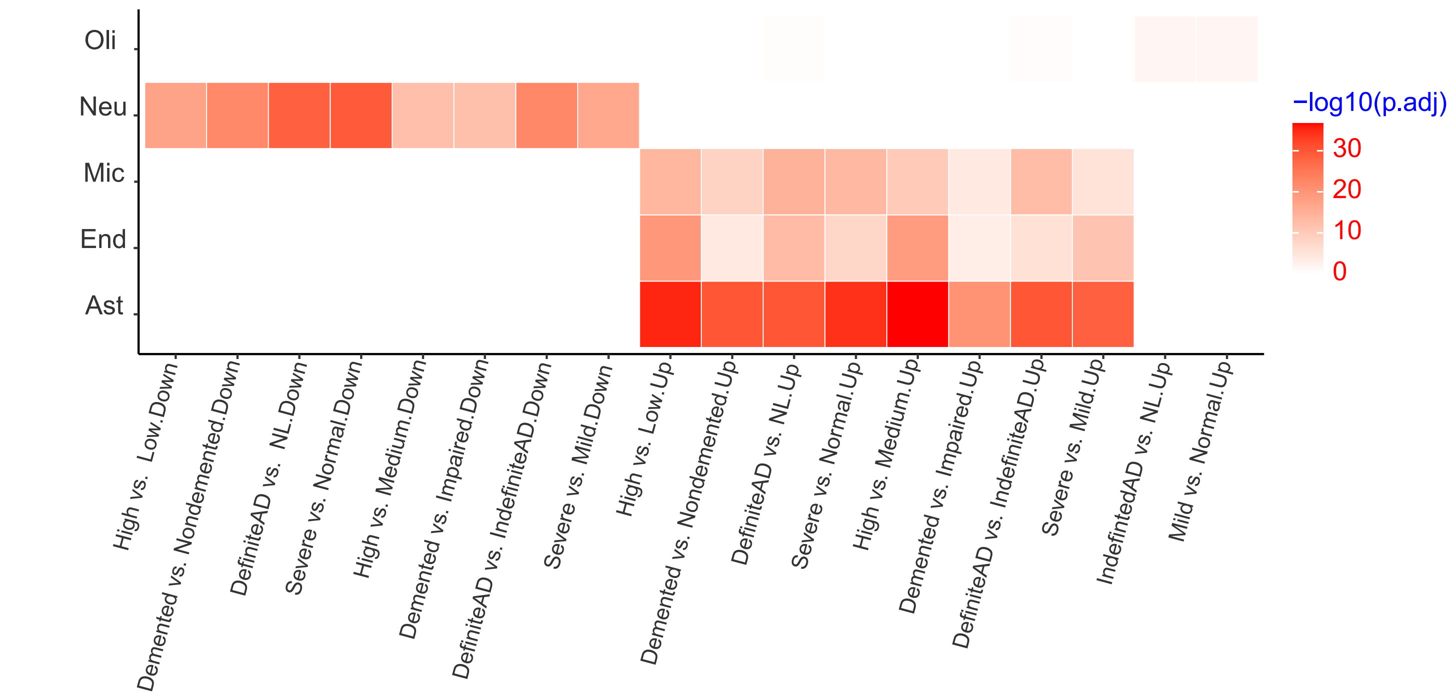

Oli
Neu
Mic
End
Ast
−log10(p.adj)
30
20
10
0
High vs. Low.Down
Demented vs. Nondemented.Down
DefiniteAD vs. NL.Down
Severe vs. Normal.Down
High vs. Medium.Down
Demented vs. Impaired.Down
DefiniteAD vs. IndefiniteAD.Down
Severe vs. Mild.Down
High vs. Low.Up
Demented vs. Nondemented.Up
DefiniteAD vs. NL.Up
Severe vs. Normal.Up
High vs. Medium.Up
Demented vs. Impaired.Up
DefiniteAD vs. IndefiniteAD.Up
Severe vs. Mild.Up
IndefintedAD vs. NL.Up
Mild vs. Normal.Up

B

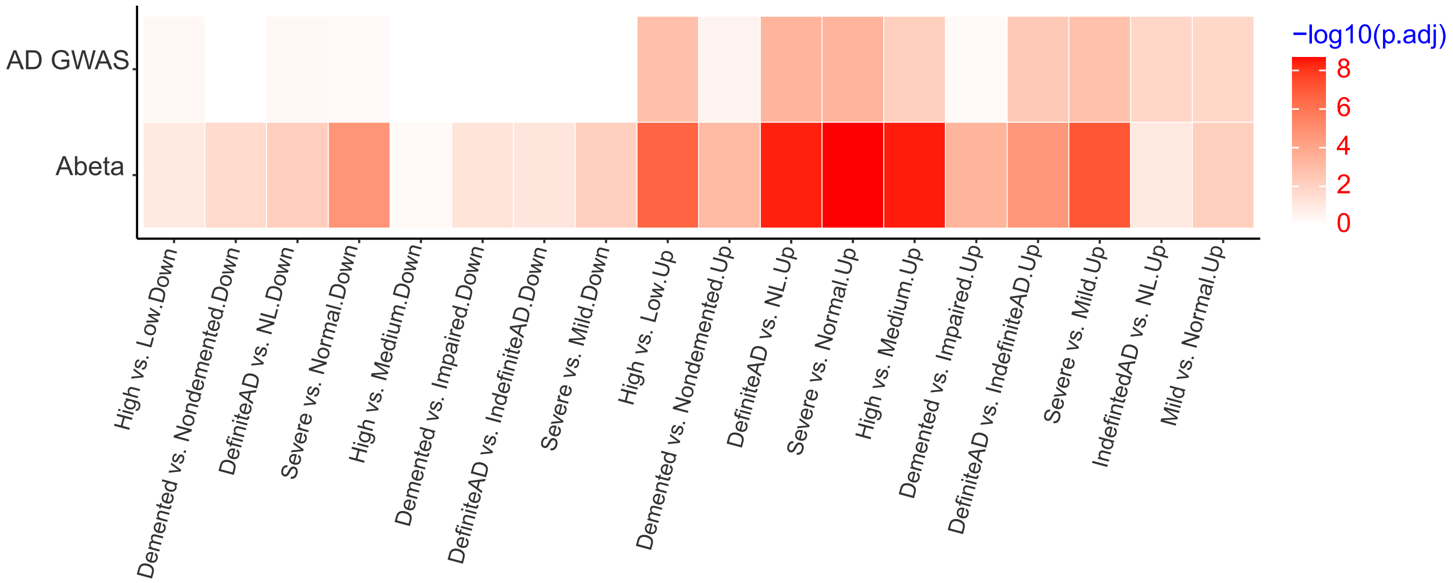

AD GWAS
Abeta
−log10(p.adj)
8
6
4
2
0
High vs. Low.Down
Demented vs. Nondemented.Down
DefiniteAD vs. NL.Down
Severe vs. Normal.Down
High vs. Medium.Down
Demented vs. Impaired.Down
DefiniteAD vs. IndefiniteAD.Down
Severe vs. Mild.Down
High vs. Low.Up
Demented vs. Nondemented.Up
DefiniteAD vs. NL.Up
Severe vs. Normal.Up
High vs. Medium.Up
Demented vs. Impaired.Up
DefiniteAD vs. IndefiniteAD.Up
Severe vs. Mild.Up
IndefintedAD vs. NL.Up
Mild vs. Normal.Up

Data S1, page 2. The enrichment of the differentially expressed proteins from the MSBB cohort for the human celltype and the AD genome-wide association study (GWAS) genetic signatures

(A) The enrichment for human brain cell-type signatures[59]. y-axis, the human cell-type signatures. Oli, oligodendrocytes; Neu, neurons, Mic, microglia; End, endothelial cells; Ast, astrocytes.

(B) The enrichment for AD GWAS and amyloid-β (aβ) genetic signatures[41]. y-axis, the AD GWAS and aβ genetic signatures. Abeta, aβ.

For (A) and (B), only DEP signatures with significantly enriched for human brain celltype and AD GWAS or aβ genetic signatures are shown. The "Up" and "Down" after '.' in a contrast denotes up- and down-regulated DEPs, respectively. x-axis, DEP signatures. The legend is the –log10(p.adj): the higher the value the more enriched.

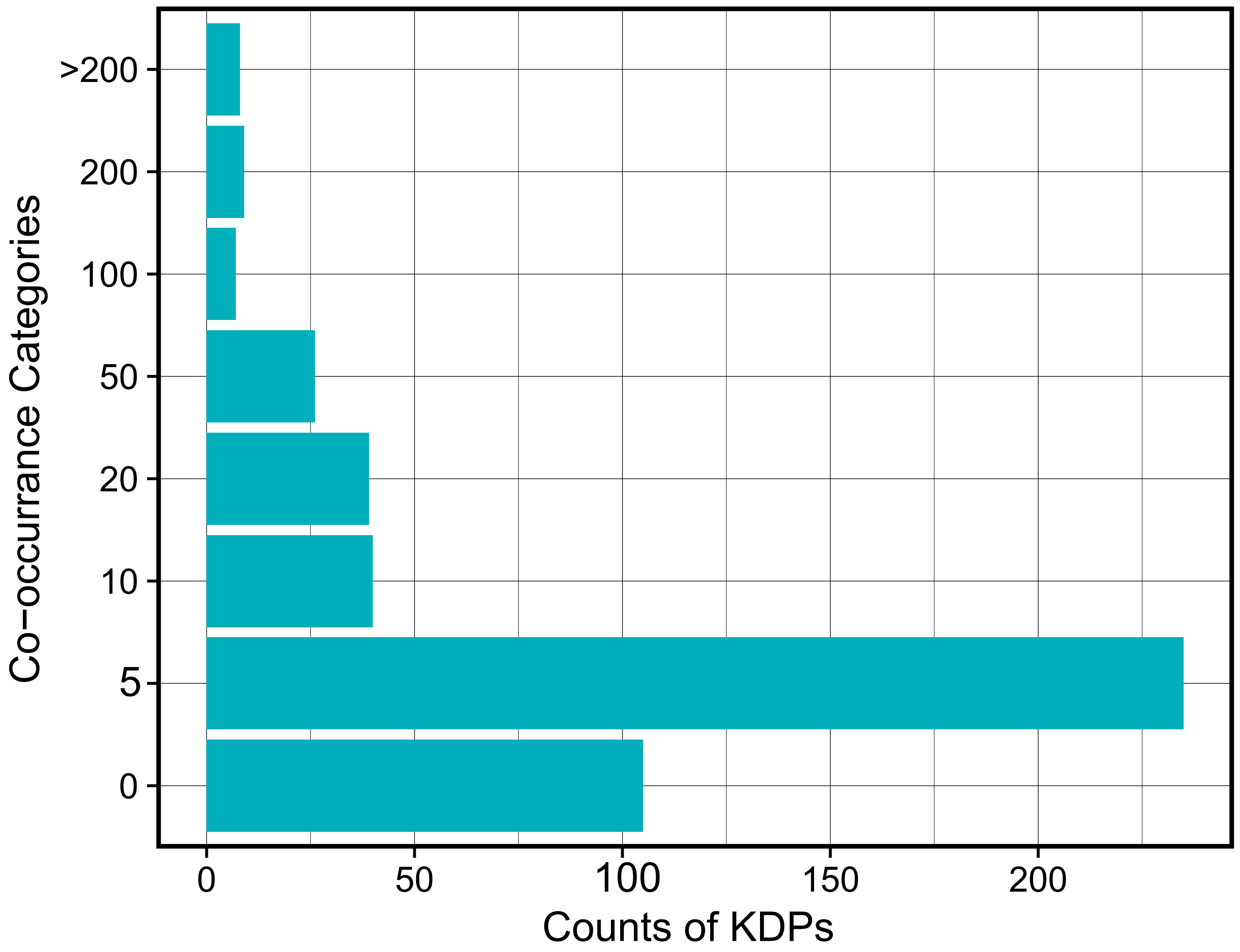

Co−occurrance Categories
>200
200
100
50
20
10
5
0
0
50
100
150
200
Counts of KDPs

Data S1, page 3. Summary of the numbers of papers where individual KDPs and Alzheimer's disease co-occur

y-axis, the groups of co-occurrence. 0, no overlap; 5, 5 ≥ number of overlap > 0; 10, 10 ≥ number of overlap > 5; 20, 20 ≥ number of overlap > 10; 50, 50 ≥ number of overlap > 20; 100, 100 ≥ number of overlap > 50; 200, 200 ≥ number of overlap > 100; and >200, number of overlap > 200. x-axis, counts of papers in each category group.

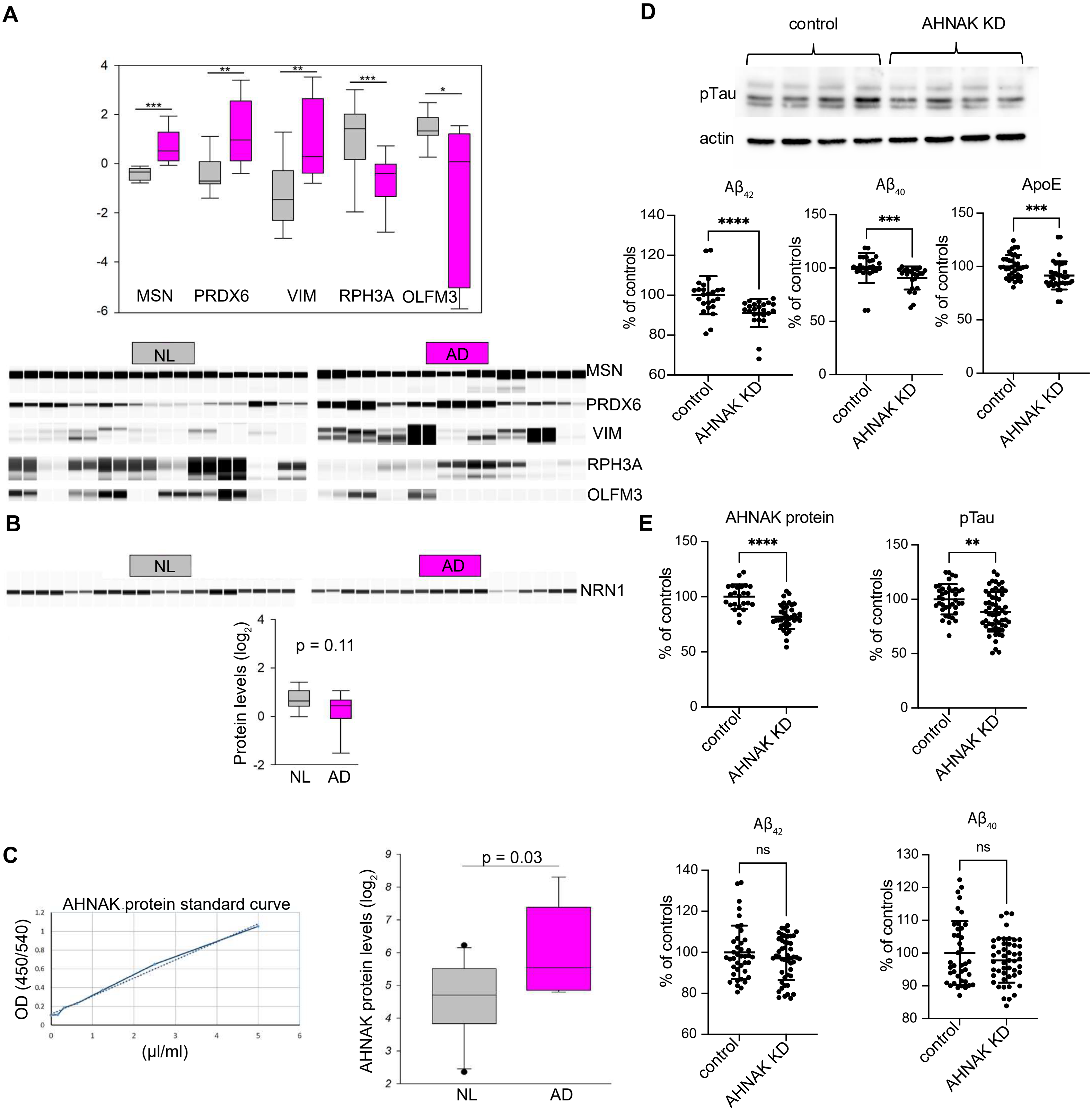
A
4
2
0
-2
-4
-6
***
**
**
***
*
MSN PRDX6 VIM RPH3A OLFM3
NL
AD
MSN
PRDX6
VIM
RPH3A
OLFM3
B
NL
AD
NRN1
Protein levels (log2)
p = 0.11
NL AD
C
AHNAK protein standard curve
OD (450/540)
(µl/ml)
AHNAK protein levels (log2)
p = 0.03
NL AD
D
control
AHNAK KD
pTau
actin
Aβ42
Aβ40
ApoE
% of controls
****
***
***
control
AHNAK KD
E
AHNAK protein
pTau
****
**
Aβ42
Aβ40
ns
ns

Data S1, page 4. Experimental and functional validation of key driver proteins (KDP) for the MSBB cohort, related to Figure 7

(A - C) Western blot validation of the expression of top-ranked KDPs in human postmortem brain PHG tissue**.** Validations of the key driver proteins changes in PHG of individuals with DefiniteAD (N=9) and NL controls (N=10). (A), a box plot of 5 KDPs with the corresponding capillary Western images. (B), box plot and Western image of NRN1. Samples were loaded in duplicates. (C), box plot and standard curve of AHNAK protein measured by ELISA. *** $p \leq 0.001$; ** $p \leq 0.01$; * $p \leq 0.05$.

(D) Down-regulation of AHNAK in *APOE44* human iPSC-derived astrocyte culture. Top panel, representative western blot images of pTau (detected by AT270) and actin in *APEO4*4 human-iPSC derived astrocytes in the presence of scramble (control) or AHNAK shRNA (AHNAK KD) lenti-virus treatment. Bottom panels, levels of human $A\beta_{42}$, $A\beta_{40}$ and APOE measured in media of astrocytes with or without AHNAK down-regulation. N=24-36/condition. Results were presented as % of controls with levels in iPSC-derived astrocytes from samples treated with scramble ctrl conditions as 100%. ****$p<0.0001$; ***$p<0.001$ with independent-samples t-test.

(E) Co-culture of mouse primary cortical neurons from 5xFAD mice with *APOE44* human iPSC-derived astrocytes treated with scramble (control) or AHNAK shRNA (AHNAK KD) virus conditions. Top panel: AHNAK protein and pTau levels (detected by AT270) in cell lysates. N=24-58/condition. Bottom panels: Human $A\beta_{42}$ and $A\beta_{40}$ levels measured in media of co-cultures. N=24-58/condition. Results were presented as % of controls with levels in co-culture samples treated with scramble ctrl conditions as 100%. ****$p<0.0001$; **$p<0.01$ with independent-samples t-test.

See also Table S6, pages 1, 3-6, and 7-11.

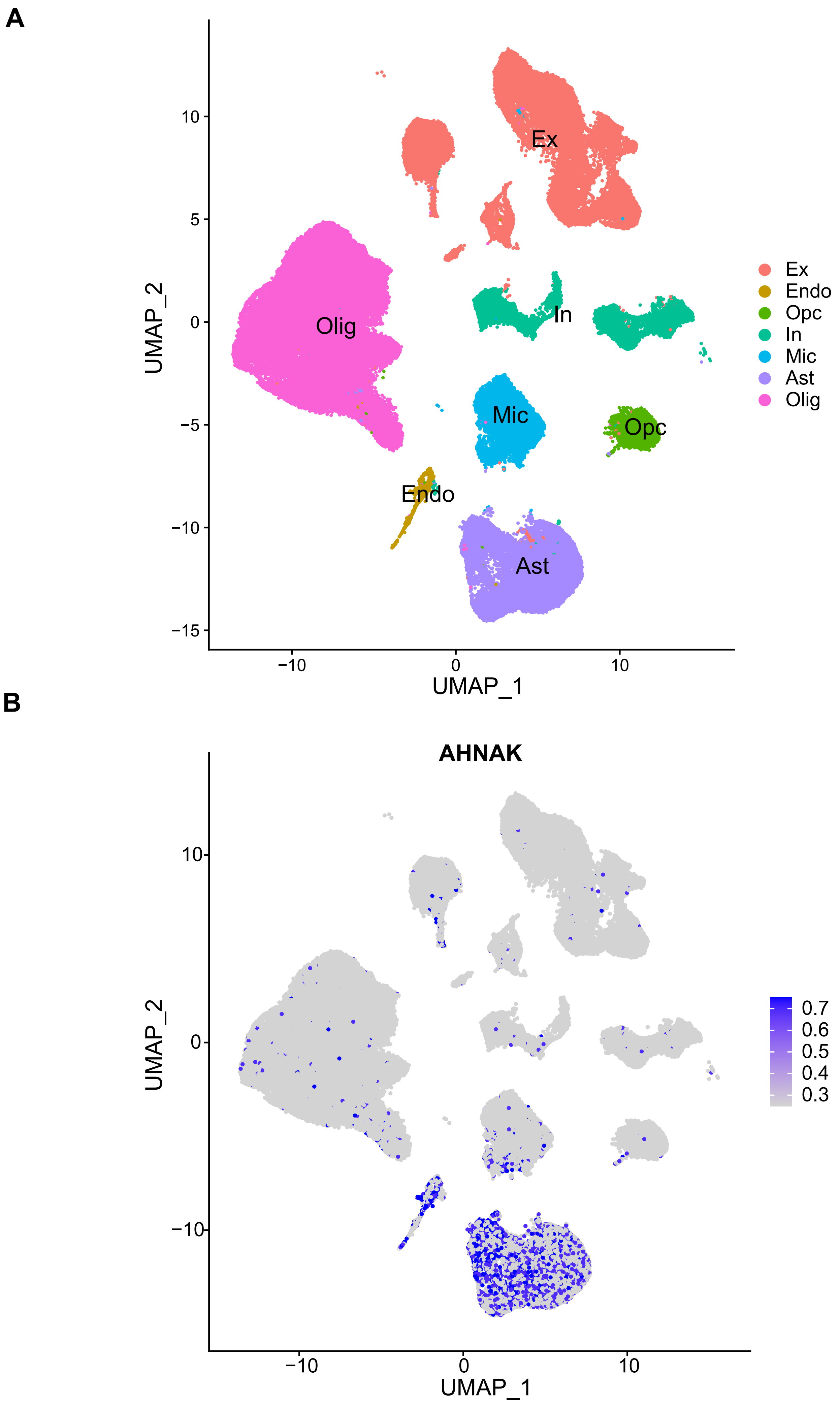
A
Ex
Olig
In
Mic
Opc
Endo
Ast
UMAP_2
UMAP_1
Ex
Endo
Opc
In
Mic
Ast
Olig
B
AHNAK
UMAP_2
UMAP_1
0.7
0.6
0.5
0.4
0.3

Data S1, page 5. Astrocyte-specific expression of the AHNAK gene in the human brain PFC
Left panel, the joint UMAP of 100,000 nuclei across 7 major brain celltype including excitatory neurons (Ex), inhibitory neurons (In), oligodendrocytes (Olig), oligodendrocyte precursor cells (Opc), astrocytes (Ast), microglia (Mic), and endothelial cells (Endo). Right panel, feature plot to show the celltype-specific expression of the AHNAK gene. Clearly, the AHNAK is primarily expressed in the astrocytes.

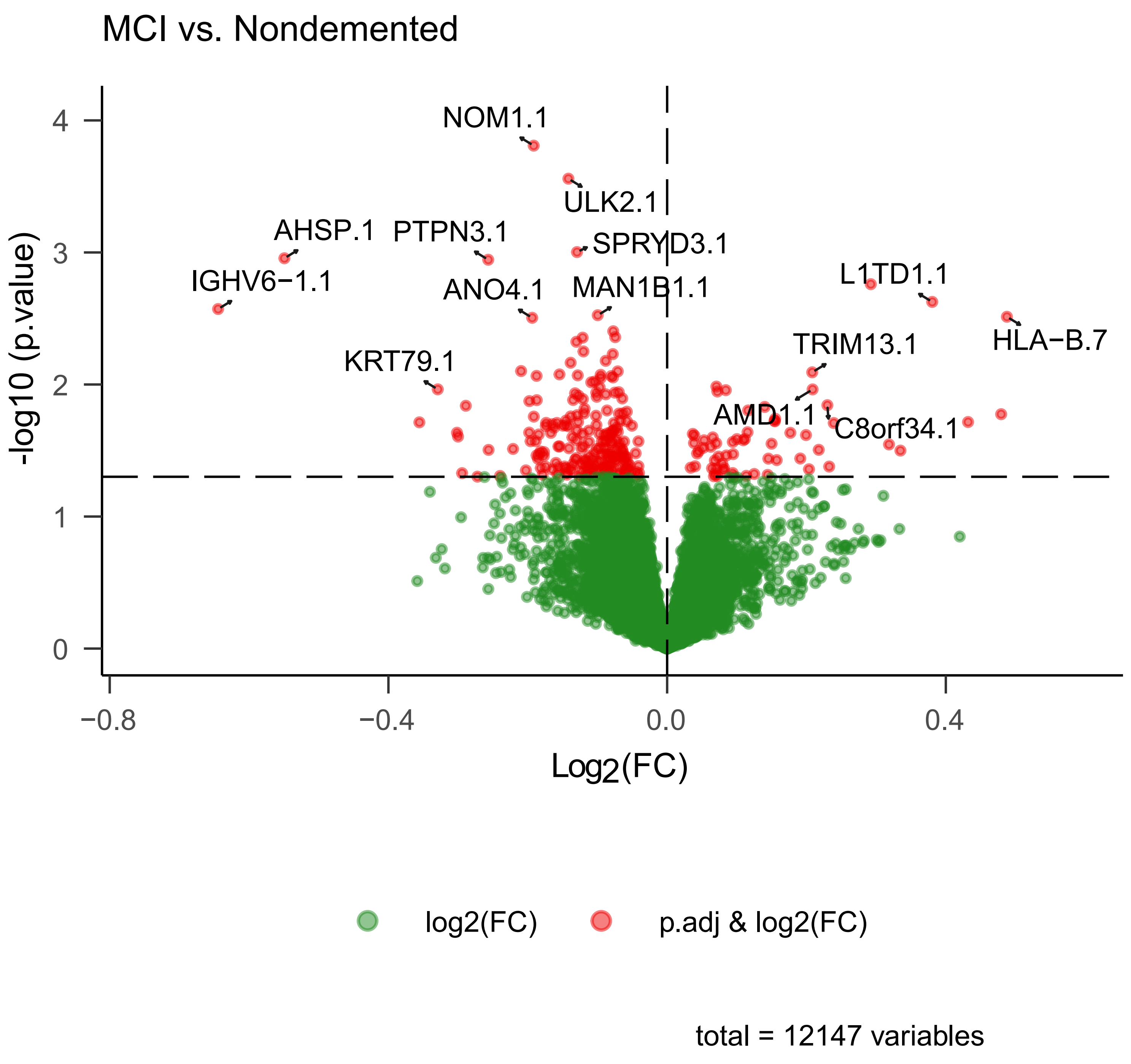
MCI vs. Nondemented
NOM1.1
ULK2.1
AHSP.1
PTPN3.1
SPRYD3.1
IGHV6−1.1
ANO4.1
MAN1B1.1
L1TD1.1
HLA−B.7
TRIM13.1
KRT79.1
AMD1.1
C8orf34.1
-log10 (p.value)
Log2(FC)
4
3
2
1
0
−0.8
−0.4
0.0
0.4
log2(FC)
p.adj & log2(FC)
total = 12147 variables

Data S1, page 6. Volcano plot showing proteins that were differentially expressed in the PHG region in MCI vs. Nondemented subjects

Note, each dot represents a distinct protein, and highlighted are the top-ranked DEPs with the lowest p value. The number after '.' in a gene symbol denotes different protein isoforms. Dots in red and green stand for DEPs with significant (p value < 0.05) and insignificant difference, respectively.
