# Supplementary material for: Multiscale proteomic modeling reveals protein networks driving Alzheimer’s disease pathogenesis
Source: Cell. Author manuscript; Available in PMC 2026 Jan 28. (PMC12851831; doi:10.1016/j.cell.2025.08.038)
Supplement: 8 [file NIHMS2129767-supplement-8.pdf]

CellPress
OPEN ACCESS

Supplemental figures

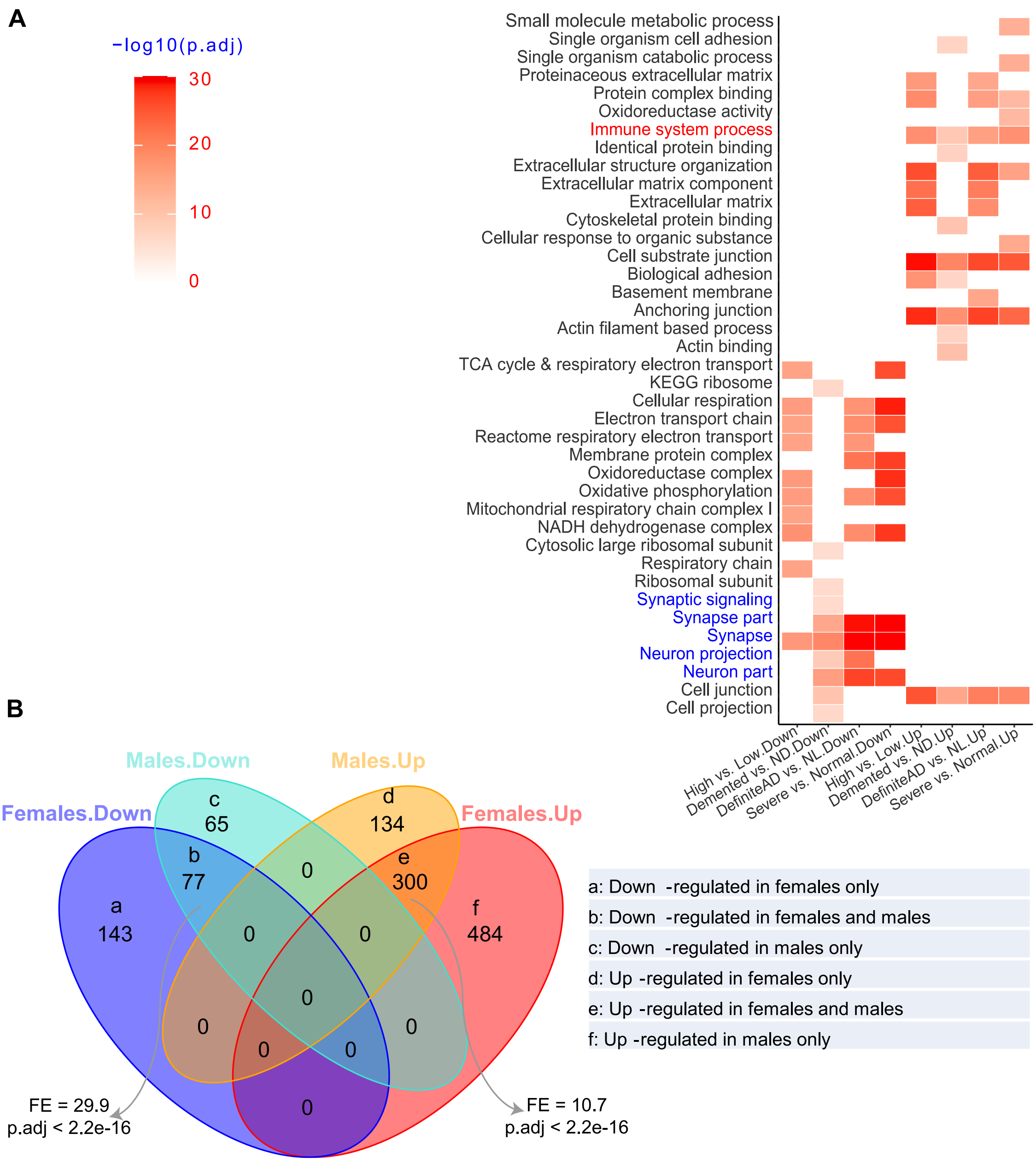

Figure S1. Biological significance of DEPs and sex differences in the PHG of the MSBB cohort, related to Figure 2

(A) Heatmap to show the enrichment of GO for the DEP signatures from Figure 2C. Only the top 10 most significantly enriched GO terms (FDR < 0.05) were shown. The “up” and “down” after “.” in a contrast denote upregulated and downregulated DEPs, respectively. Highlighted GO terms in blue and red are neuronal and immune processes, respectively.

(B) The Venn diagram showing the number of DEPs that are shared or distinct to females or males.

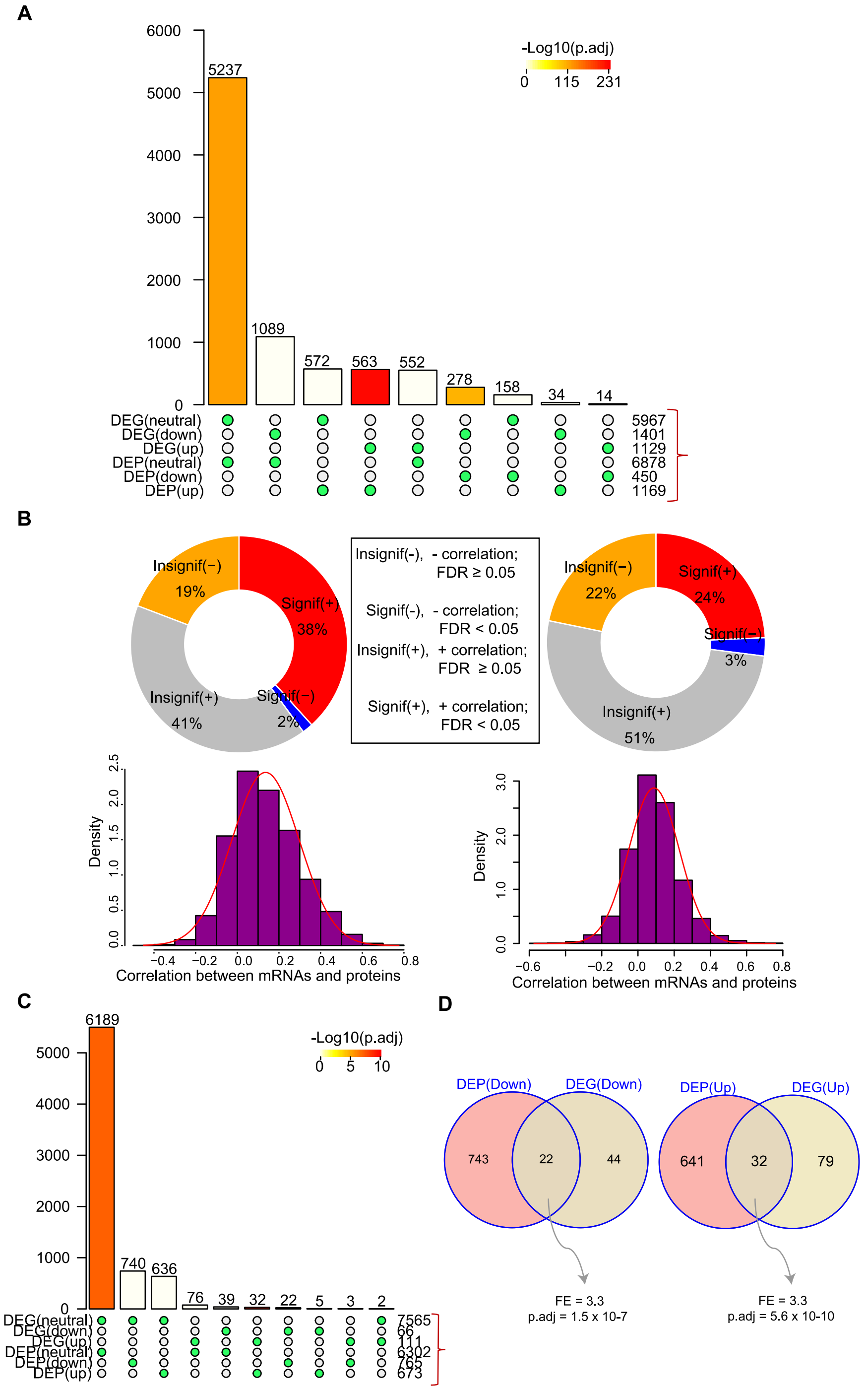

Figure S2. The analysis of concordance between DEPs and DEGs, related to Figure 3

(A) SuperExactTest of composite DEPs and DEGs from the brain region PHG of the MSBB cohort.

(B) Correlation of the expression between proteins and their coding mRNAs. Upper, the donut charts of correlation categories. Bottom, the density plots of the correlation coefficients. MSBB, left panel; ROSMAP, right panel.

(C) SuperExactTest of the composite DEGs and DEPs from the ROSMAP PFC. DEG(neutral), DEG(down), and DEG(up) denote composite DEG unchanged, decreased, and increased in AD vs. NL, respectively.

(D) Venn diagrams to show the concordance between DEPs and DEGs from the ROSMAP PFC.

See also Table S3, pages 3, 4, 6, and 7.

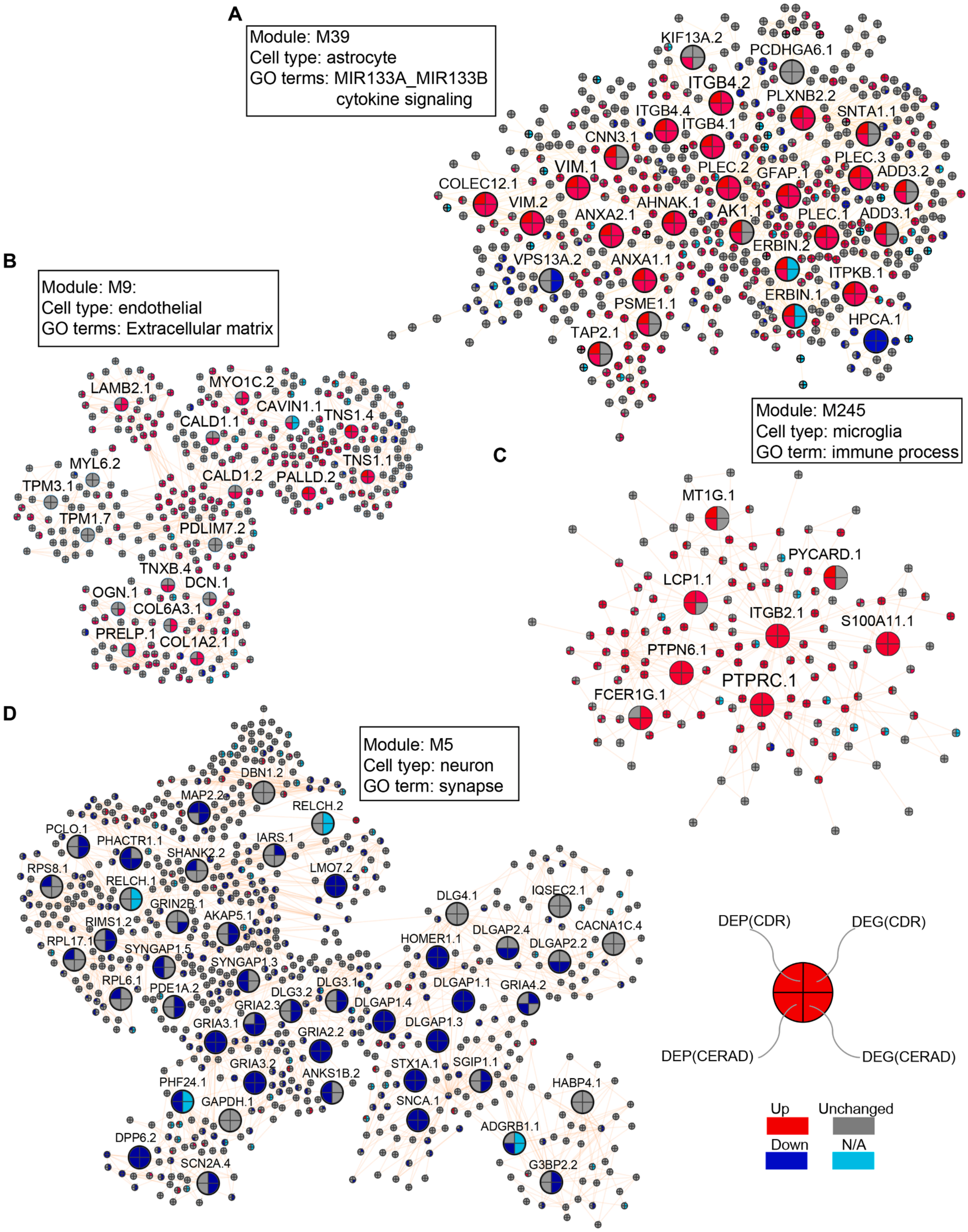

Figure S3. The representative subnetworks of the PHG proteomics MEGENA network in the MSBB cohort, related to Figure 4

(A) Module M39.

(B) Module M9.

(C) Module M245.

(D) Module M5.

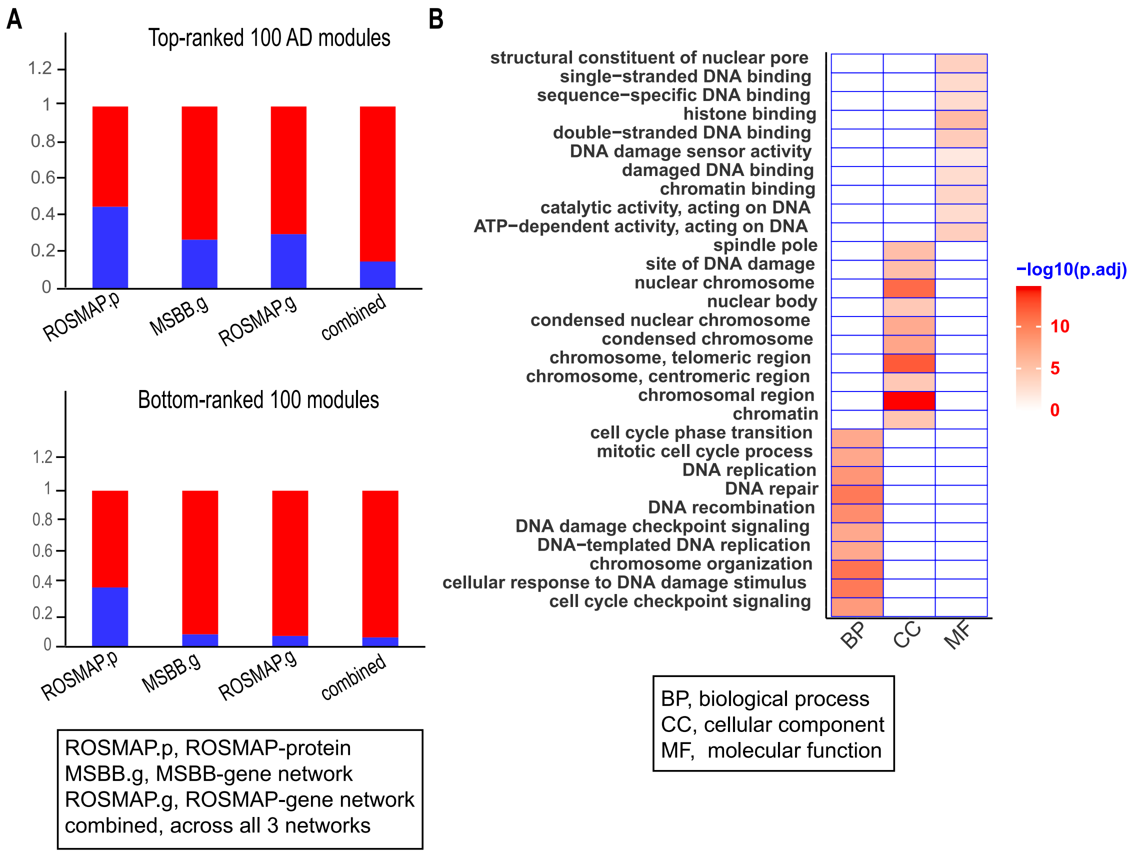

Figure S4. Summary of PHG protein module conservation across different data modalities and brain regions, related to Figure 5

(A) Bar plots showing module preservation for the top- and bottom-PHG protein modules ranked by their association with AD.

(B) GO enrichment for the gene set shared by M10 and the gene signature of MG12.

CellPress
OPEN ACCESS

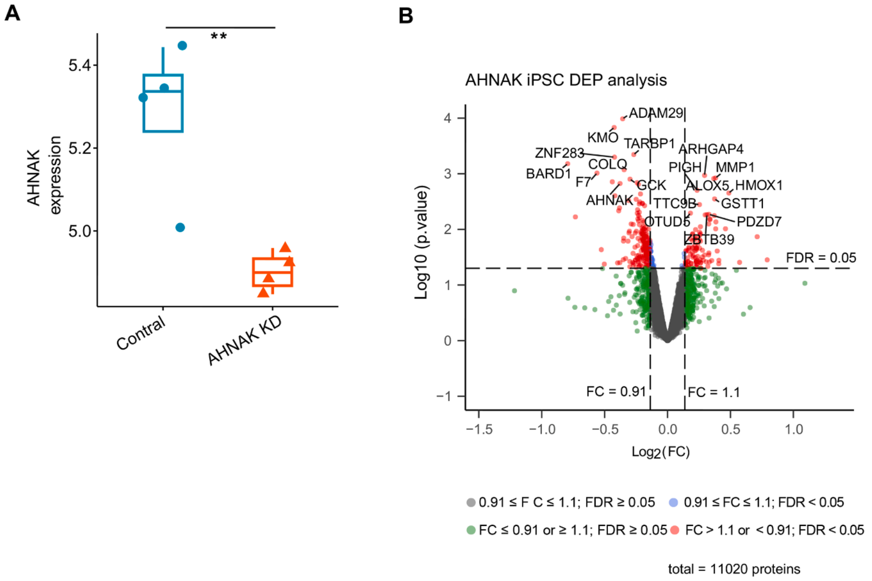

Figure S5. Proteomic profiling of human iPSC-derived astrocytes with *AHNAK* KD, related to Figure 7

(A) Boxplot showing the AHNAK expression in the proteome of the *APOE* 44 AD human iPSC-derived astrocytes treated with *AHNAK* shRNA (KD) or scramble-shRNA (control).

(B) Volcano plot showing DEPs upon *AHNAK* KD in *APOE* 44 AD human iPSC-derived astrocytes.
